# Dose-response relationship of pre-ICU corticosteroids and influenza-associated pulmonary aspergillosis: a multicenter target trial emulation

**DOI:** 10.1016/j.aicoj.2026.100132

**Published:** 2026-08-03

**Authors:** Shengnan Xu, Hongmei Liu, Pengli Fan, Hao Dou, Leilei Guo, Bo Qiao, Yuanyuan Li, Yansheng Zhou, Xiufeng Hu, Yanyan Guo, Bin Hu, Sangang Zheng, Junping Liu, Jiangfeng Zhang, Junzhe Bao, Peng Li

**Affiliations:** a Respiratory Intensive Care Unit, Henan Provincial People’s Hospital, People’s Hospital of Zhengzhou University, Zhengzhou, China; b Department of Pharmacy, Henan Provincial People’s Hospital, People’s Hospital of Zhengzhou University, Zhengzhou, China; c Department of Respiratory and Critical Care Medicine, Pulmonary Endoscopy Center, Henan Provincial People’s Hospital, People’s Hospital of Zhengzhou University, Zhengzhou, China; d Infection Prevention and Control Department, Zhengzhou Central Hospital Affiliated to Zhengzhou University, Zhengzhou, China; e Infection Prevention and Control Department, Henan Provincial Chest Hospital, Zhengzhou, China; f Infection Prevention and Control Department, The First Affiliated Hospital of Nanyang Medical College, Nanyang, China; g Infection Prevention and Control Department, The First Affiliated Hospital of Henan Medical University, Xinxiang, China; h Infection Prevention and Control Department, The First Affiliated Hospital of Henan University of Science and Technology, Luoyang, China; i Infection Prevention and Control Department, Huaihe Hospital of Henan University, Kaifeng, China; j Infection Prevention and Control Department, Zhumadian Central Hospital, Zhumadian, China; k Infection Prevention and Control Department, Jiyuan People's Hospital, Jiyuan, China; l Department of Infectious Disease, Henan Provincial People’s Hospital, People’s Hospital of Zhengzhou University, Zhengzhou, China; m Department of Clinical Microbiology, Henan Provincial People’s Hospital, People’s Hospital of Zhengzhou University, Zhengzhou, China; n College of Public Health, Zhengzhou University, Zhengzhou, China; o Department of Infection Control, Henan Provincial People’s Hospital, People’s Hospital of Zhengzhou University, Zhengzhou, China

**Keywords:** Influenza-associated pulmonary aspergillosis, Corticosteroid, Dose-response, Target trial emulation, Directed acyclic graphs

## Abstract

**Background:**

Prior corticosteroid use is linked to influenza-associated pulmonary aspergillosis (IAPA) in critically ill patients, yet the dose-response relationship remains unclear.

**Methods:**

In this multicenter emulated target trial across 48 ICUs in Central China (January 2023-June 2025), we included critically ill, non-neutropenic adults with influenza. Using inverse probability of treatment weighting (IPTW) and restricted cubic splines, we modeled the dose-response association between pre-ICU corticosteroid exposure and IAPA, identifying risk thresholds via segmented regression. Effect modification by pre-specified patient characteristics was further assessed.

**Results:**

Among 2763 patients (74.2% exposed to corticosteroids; median dose 35 mg dexamethasone-equivalent), 191 (6.9%) developed IAPA during their ICU stay. Pre-ICU corticosteroid exposure was independently associated with IAPA (weighted HR = 2.11; 95% CI 1.25–3.56). A non-linear, J-shaped dose-response relationship was identified (P < 0.001). Risk increased continuously, with a 50% increase at 43.4 mg and doubling at 51.4 mg, followed by a steep inflection point at 63.7 mg (95% CI 62.8−64.7). Higher SOFA scores (>7) significantly amplified per-dose risk (P < 0.001), lowering thresholds to 35.2 mg for 50% increase and 39.5 mg for doubling.

**Conclusions:**

Pre-ICU corticosteroid exposure is associated with a nonlinear, dose-dependent increase in IAPA risk. Cumulative doses approaching 40−50 mg warrant heightened vigilance, particularly in severely ill patients. These findings support individualized, dose-aware corticosteroid stewardship in severe influenza.

## Background

In critically ill patients, influenza is frequently complicated by secondary infections, among which influenza-associated pulmonary aspergillosis (IAPA) is particularly lethal, with an attributable mortality approaching 50% [[1], [2], [3]]. The convergence of viral-induced immune dysregulation and epithelial damage creates a permissive environment for Aspergillus [4]. Several established risk factors for IAPA have been identified, including male sex, smoking history, chronic lung disease, immunosuppressive conditions (e.g., haematological malignancy and solid organ transplantation), and influenza A (H1N1) subtype [2,3]. Corticosteroids, while commonly used to mitigate pulmonary inflammation, remain a double-edged sword in this context [5,6]. Although previous meta-analyses have identified pre-ICU corticosteroid use as a risk factor for IAPA [2,3,7], critical gaps persist: the shape of the dose-response relationship, the existence of a safe threshold, and patient-level susceptibility modifiers remain undefined [8,9]. To address these gaps, we emulated the design of a hypothetical target trial in a multicenter cohort of critically ill, non-neutropenic influenza patients. We aimed to: (1) investigate the association between pre-ICU corticosteroid initiation and IAPA; (2) characterize the dose-response curve and identify clinically actionable risk thresholds; and (3) assess effect modification by baseline patient characteristics. By quantifying these thresholds, we provide evidence to guide individualized, dose-aware corticosteroid prescribing.

## Methods

### Study design

This multicenter cohort study emulated a target trial across 48 ICUs in nine tertiary hospitals in Central China. Clinical data were extracted from Hospital and Laboratory Information Systems (HIS/LIS) using standardized case report forms. The study protocol was approved by the ethics committees of all participating hospitals (lead registry number: HNSRY2023-37). Given the non-interventional, observational design and the use of fully anonymized patient data, the requirement for individual informed consent was waived.

### Eligibility criteria

**Fig. 1:**
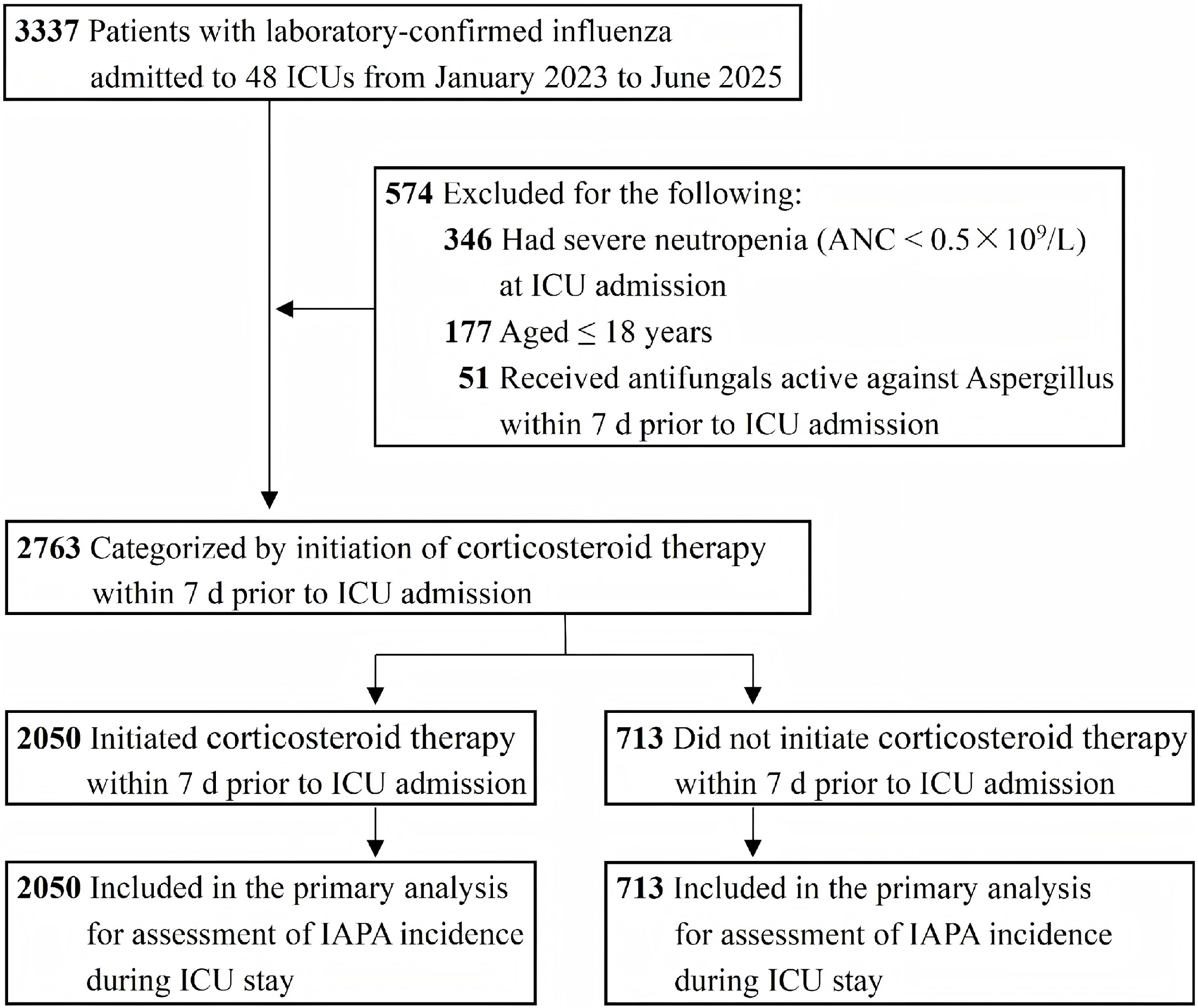
Study cohort and emulated trial flow.

We included adult patients (aged ≥18 years) with RT-PCR-confirmed influenza admitted to the participating ICUs between January 2023 and June 2025. Patients were excluded if they: (1) were aged <18 years; (2) had neutropenia at ICU admission (absolute neutrophil count [ANC] <0.5 × 10⁹/L); or (3) received antifungal agents active against Aspergillus within 7 days prior to ICU admission (Fig. 1). The neutropenia threshold (ANC <0.5 × 10⁹/L) was applied to exclude patients with this well-established, profound risk factor for invasive aspergillosis [10], thereby isolating the effects of corticosteroid exposure and influenza-associated immunopathology in a non-neutropenic critically ill population.

### Emulated intervention strategies

We defined two hypothetical intervention strategies: (1) initiation or continuation of systemic corticosteroids during the 7 days preceding ICU admission, and (2) avoidance of any systemic corticosteroid during the same period. Exposure was defined as receipt of any therapeutic dose of a systemic corticosteroid (e.g., prednisone, methylprednisolone, hydrocortisone, or dexamethasone) within this 7-day window, encompassing both new initiations and continuation of pre-existing chronic therapy (e.g., for COPD, asthma, or autoimmune conditions). The cumulative dose was quantified as the total dexamethasone-equivalent administered during this period. In accordance with the intention-to-treat (ITT) principle, exposure status was determined based on pre-ICU corticosteroid use and fixed at ICU admission for the primary analysis, regardless of subsequent in-ICU corticosteroid administration or dose adjustments. Under the TTE framework, we defined time zero as the moment of ICU admission, at which eligibility assessment, treatment strategy assignment (based on the 7-day pre-ICU exposure assessment window), and follow-up initiation occurred simultaneously; the pre-ICU 7 days served solely as the exposure assessment window to establish baseline assignment, not as an alternative time zero or a modification of the follow-up start. Details on pre-ICU corticosteroid indications, exposure timeline, and protocol components are provided in eTable S1, Figure S1, and Table S2, respectively.

### Follow-up and outcomes

The primary outcome was incident proven or probable IAPA during the ICU stay, defined according to the 2020 consensus criteria of the European Confederation of Medical Mycology and the International Society for Human and Animal Mycology (ECMM/ISHAM) [11]. The diagnostic workup for IAPA, including bronchoscopy, bronchoalveolar lavage (BAL), galactomannan (GM) testing in serum and BAL fluid, Aspergillus PCR, fungal cultures, and chest CT imaging, was not standardized across all participating centers and was performed at the discretion of the treating physicians. The time zero was defined as the moment of ICU admission, which corresponds to the point of treatment assignment in the hypothetical randomized trial. Follow-up began at time zero and ended at the earliest of ICU discharge, death, or the database closure date (June 30, 2025).

### Statistical analysis

The sample size was determined by the availability of data from the defined cohort period. We constructed directed acyclic graphs (DAG) informed by (1) a systematic review of PubMed- and EMBASE-indexed literature through June 2025 and (2) structured expert consultations using the Delphi method, to identify potential confounders of the association between pre-ICU corticosteroid exposure and IAPA, and to avoid selection bias or over-adjustment bias in the subsequent estimation of this effect.

Based on the minimal sufficient adjustment set identified through d-separation criteria in the DAG, we employed inverse probability of treatment weighting (IPTW) to balance the distribution of covariates between patients with and without pre-ICU corticosteroid exposure, emulating a randomized trial. Propensity scores were estimated using unconditional logistic regression, with pre-ICU corticosteroid exposure as the dependent variable and all DAG-identified confounders as independent variables. To account for nonlinear relationships with the exposure status, a likelihood ratio test was performed, and continuous variables that exhibited significant nonlinearity were modeled using restricted cubic splines (RCS) with three knots. After IPTW, covariate balance was assessed by calculating absolute standardized mean differences (SMDs; <0.1 considered adequate). We then fitted an IPTW-weighted Cox proportional hazards model with pre-ICU corticosteroid exposure as the sole independent variable to estimate its effect on IAPA risk. For comparison, we also present an unadjusted univariable Cox model and a multivariable Cox model adjusting for all DAG-identified confounders. Sensitivity analyses included: (1) trimming of extreme weights at the 5th and 95th percentiles in IPTW to reduce the influence of extreme propensity scores; (2) exclusion of patients with hematological malignancies and solid organ transplant recipients to mitigate potential confounding from these high-risk populations; and (3) calculation of E-values for the primary effect estimates to assess the robustness of our findings to unmeasured confounding.

In the IPTW-weighted cohort, we further modeled the cumulative corticosteroid dose-IAPA relationship using RCS with three knots. Segmented regression was used to identify the inflection point where the risk slope changed significantly, and clinically interpretable thresholds (HR = 1.5 and HR = 2.0) were derived via linear interpolation. In addition, to assess effect modification by pre-specified patient characteristics—selected based on their established clinical and pathophysiological relevance to infection risk in critically ill patients [12,13]—we tested interactions between cumulative corticosteroid dose and each potential modifier (BMI, diabetes, chronic respiratory disease, and SOFA score at ICU admission).

This study adhered to the Transparent Reporting of Observational Studies Emulating a Target Trial (TARGET) statement (see eTable S3 for the checklist). All analyses were performed using R software (version 4.4.2; R Foundation for Statistical Computing) with a two-tailed significance level of α = 0.05.

## Results

### Characteristics of cohort

Between January 2023 and June 2025, a total of 2763 critically ill, non-neutropenic adults with influenza from 48 ICUs met the eligibility criteria and were included in the cohort (Fig. 1). Among them, 2050 patients (74.2%) initiated corticosteroid therapy within 7 days prior to ICU admission, with a median total dose of 35 mg (IQR: 28−48 mg; dexamethasone equivalent), while 713 patients (25.8%) did not. The frequencies of key diagnostic procedures were similar between the two groups (eTable S4), arguing against substantial surveillance bias. The overall incidence of IAPA occurring during the ICU stay was 6.9% (n = 191; Table 1), with a median time from ICU admission to diagnosis of 3 days (IQR: 2–5 days) and with 144 (75.4%) cases diagnosed within the first 5 days of ICU stay.

**Table 1:** Baseline characteristics before and after target trial emulation.

Characteristics	Before target trial emulation				After target trial emulation^b^			
	Pre-ICU corticosteroid (n = 2050)	No pre-ICU corticosteroid (n = 713)	P value	SMD	Pre-ICU corticosteroid (n = 2682)	No pre-ICU corticosteroid (n = 2764)	P value	SMD
Demographics^a^								
Age (years), mean ± SD	60.5 ± 5.8	57.3 ± 5.6	<0.001	0.572	59.8 ± 5.9	59.5 ± 5.1	0.772	0.024
BMI, median (IQR)	22.3 (19.6, 25.1)	22.1 (18.9, 24.9)	0.086	0.068	22.2 (19.5, 25.1)	22.1 (18.9, 25.3)	0.589	0.028
Smoking	424 (20.7)	111 (15.6)	0.003	0.133	528 (19.7)	490 (17.7)	0.495	0.050
Comorbidities^a^								
Diabetes, n (%)	341 (16.6)	94 (13.2)	0.034	0.097	429 ( 16.0)	391 (14.1)	0.483	0.052
Chronic liver disease, n (%)	230 (11.2)	57 (8.0)	0.018	0.110	292 ( 10.9)	347 (12.6)	0.480	0.051
Chronic kidney disease, n (%)	225 (11.0)	41 (5.8)	<0.001	0.190	261 ( 9.7)	273 ( 9.9)	0.857	0.005
Chronic respiratory disease, n (%)	351 (17.1)	94 (13.2)	0.016	0.110	446 (16.6)	412 (14.9)	0.468	0.047
Clinical features at admission^a^								
Hemoptysis, n (%)	120 (5.9)	32 (4.5)	0.200	0.062	152 (5.7)	167 (6.0)	0.848	0.016
Respiratory rate >30/min, n (%)	1853 (90.4)	587 (82.3)	<0.001	0.237	2365 (88.2)	2336 (84.5)	0.149	0.108
Consolidation, n (%)	952 (46.4)	378 (53.0)	0.003	0.132	1294 (48.2)	1204 (43.6)	0.199	0.094
NLR, median (IQR)	6.6 (4.7, 9.2)	5.9 (4.6, 8.0)	<0.001	0.183	6.5 (4.6, 9.0)	6.3 (4.6, 8.2)	0.431	0.080
CRP(mg/L), median (IQR)	116 (83, 152)	92 (68, 117)	<0.001	0.535	111 (79, 146)	110 (75, 157)	0.903	0.003
SOFA, median (IQR)	7 (6, 8)	6 (5, 6)	<0.001	0.747	7 (6, 7)	7 (6, 7)	0.476	0.012
Bacterial co-infection, n (%)	775 (37.8)	220 (30.9)	0.001	0.147	980 (36.5)	880 (31.8)	0.193	0.099
Viral co-infection, n (%)	136(6.6)	16 (2.2)	<0.001	0.214	156 (5.8)	176 6.4)	0.779	0.023
Interventions at admission								
Antifungal prophylaxis, n (%)	308 (15.0)	125 (17.5)	0.127	0.068	NA			
Broad-spectrum antibiotic, n (%)	931(45.4)	291 (40.8)	0.037	0.093				
Interleukin-6 inhibitors, n (%)	89 (4.3)	28 (3.9)	0.715	0.021				
Invasive mechanical ventilation, n (%)	723 (35.3)	241 (33.8)	0.508	0.031				
Renal replacement therapy, n (%)	486 (23.7)	192 (26.9)	0.095	0.074				
ECMO, n (%)	58 (2.8)	21 (2.9)	0.976	0.007				
Others								
Admission source, n (%)			0.873	0.036	NA			
Emergency	744 (36.3)	269 (37.7)						
Community	316 (15.4)	112 (15.7)						
General wards	557 (27.2)	189 (26.5)						
Other hospitals	433 (21.1)	143 (20.1)						
Admission season, *n* (%)			0.749	0.048				
Spring	432 (21.1)	137 (19.2)						
Summer	108 (5.3)	37 (5.2)						
Autumn	205 (10.0)	71 (10.0)						
Winter	1305 (63.7)	468 (65.6)						
Time from symptom onset to ICU (days), median (IQR)	3 (2, 4)	3 (2, 4)	0.374	0.035				

Abbreviations: SD:standard deviation; BMI: body mass index; IQR: interquartile range; NLR: neutrophil-to-lymphocyte ratio; CRP: C-reactive protein; ECMO: extracorporeal membrane oxygenation; SOFA: sequential organ failure assessment score; SMD: absolute standardized mean difference.

a Confounders identified by DAGs and included in the propensity score model.

b The sample sizes shown for the corticosteroid and no-corticosteroid groups represent the effective sample sizes in the weighted pseudo-population after IPTW and differ from the original cohort due to the weighting procedure.

### Causal inference and IPTW balancing

**Fig. 2:**
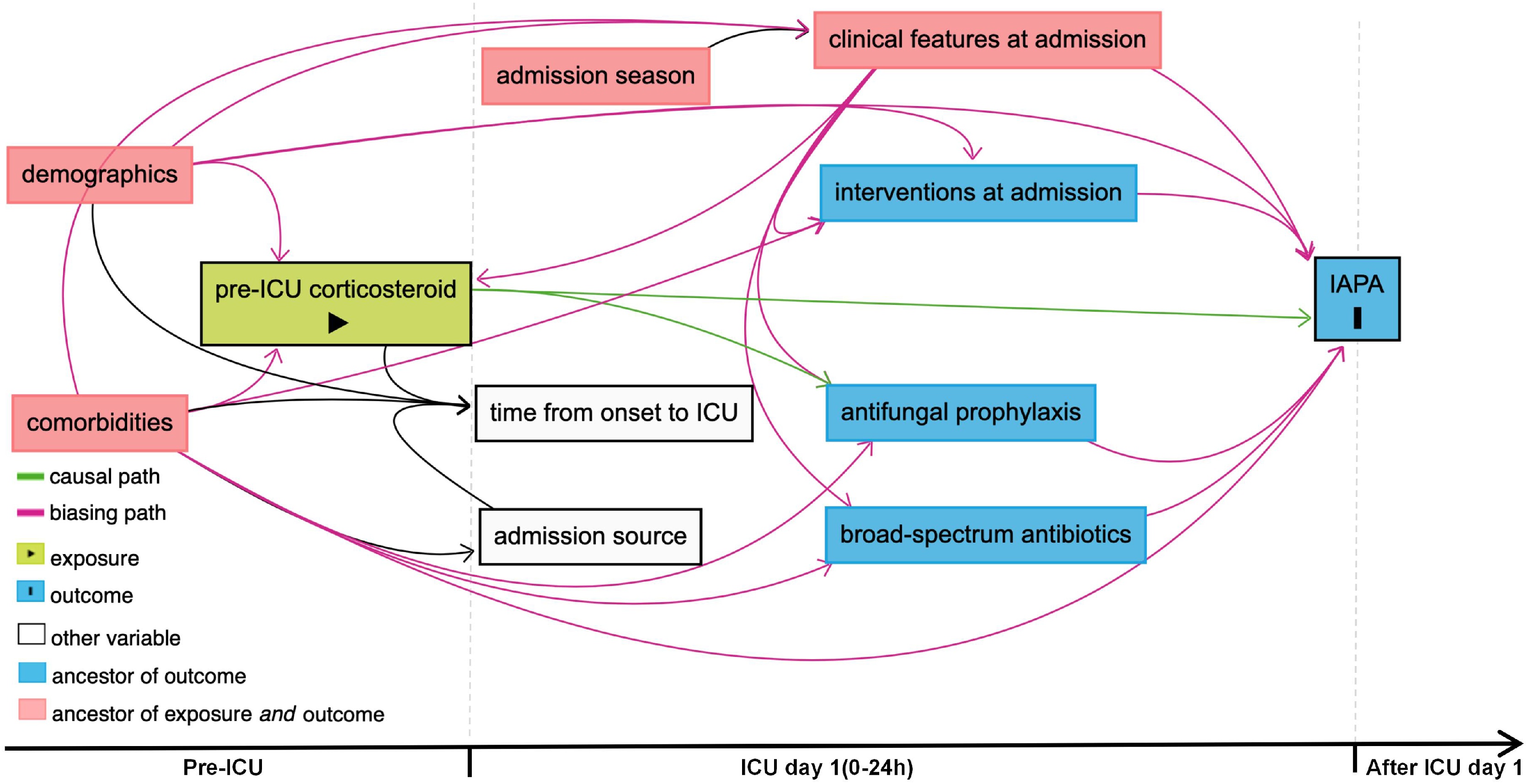
DAG framework for the effect of pre-ICU corticosteroid initiation on the occurrence of IAPA.

Based on the predefined DAG (Fig. 2, eTable S5), a minimal sufficient adjustment set comprising 15 key confounders across three domains—demographics, comorbidities, and clinical features at ICU admission—was incorporated into the propensity score model for pre-ICU corticosteroid exposure. To account for nonlinear relationships with the exposure status, CRP and SOFA score at ICU admission were modeled using RCS with three knots (P for nonlinear <0.001 for both). After applying IPTW, excellent balance was achieved between the corticosteroid-exposed and non-exposed groups. All covariates had absolute SMD < 0.1, with the exception of respiratory rate (SMD = 0.108, P = 0.149) (Table 1, eFig. S2 and S3). The sample size after weighting was 5446, representing the effective size of the weighted pseudo-population, with 2682 (49.2%) patients in the corticosteroid-exposed group.

### Relationship between pre-ICU corticosteroid exposure and IAPA risk

**Fig. 3:**
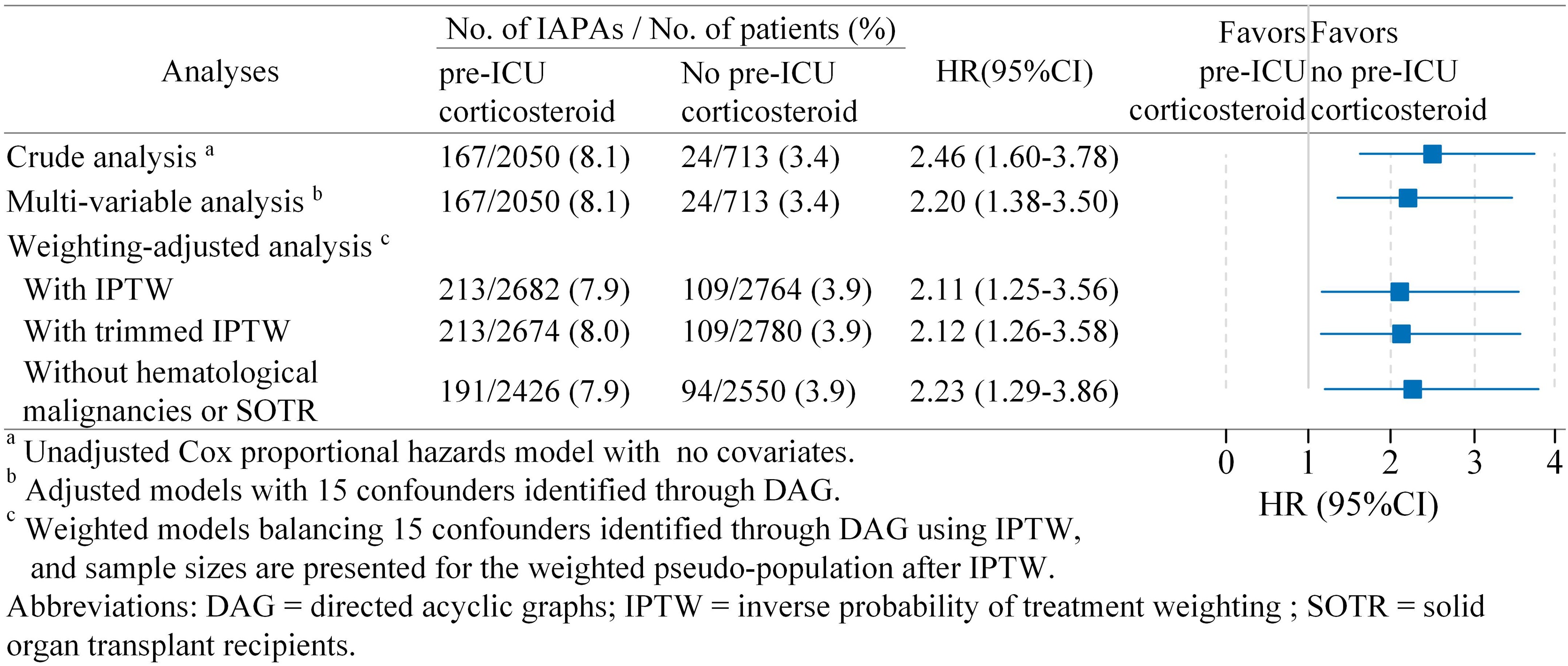
Association of pre-ICU corticosteroid initiation with IAPA risk.

Pre-ICU corticosteroid exposure (binary) was consistently associated with increased IAPA risk across models, with progressive attenuation after adjustment (Fig. 3). In the IPTW-weighted model, the HR was 2.11 (95% CI 1.25–3.56), further attenuated from the multivariable-adjusted estimate (HR = 2.20) in which BMI, NLR, CRP, and SOFA score were modeled using RCS due to their significant nonlinearity with IAPA status (P for nonlinearity <0.001 for each). In sensitivity analyses, after trimming extreme weights, the HR was 2.12; after excluding patients with hematological malignancies and solid organ transplant recipients, the adjusted HR was 2.23; the E-value for the primary adjusted HR was 3.64, suggesting that unmeasured confounding would need to be of substantial magnitude to fully explain the observed association (eFig. S4).

**Fig. 4:**
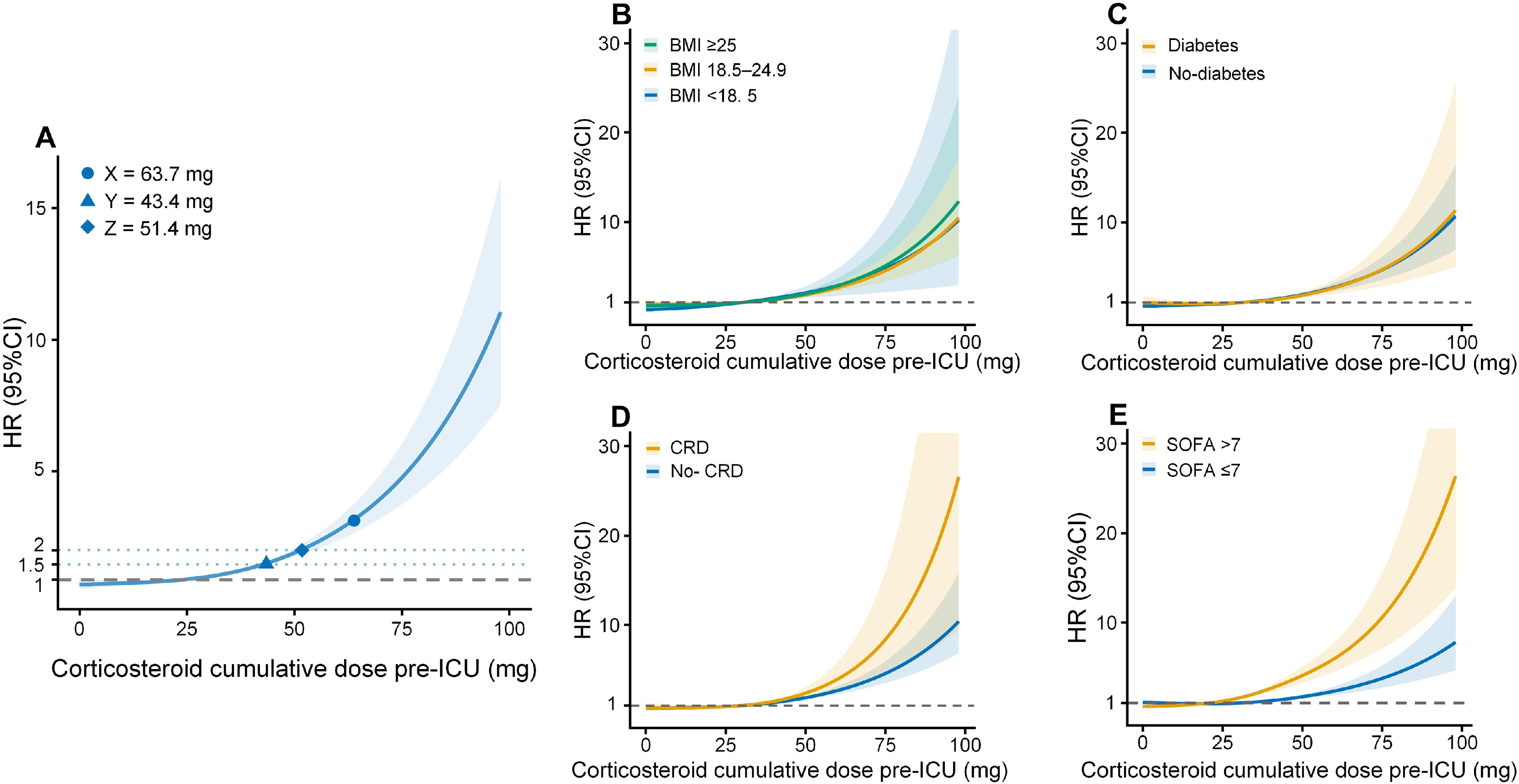
Overall and stratified dose-response relationships between pre-ICU corticosteroid dose and IAPA risk (Panel A shows the dose-response curve in the overall IPTW-weighted cohort. Panels B-E display the curves stratified by BMI, diabetes, chronic respiratory disease, and SOFA score at ICU admission, respectively.).

Modeling cumulative pre-ICU corticosteroid dose as a continuous variable revealed a significant nonlinear, J-shaped association (P for nonlinearity <0.001; Fig. 4A). Risk increased gradually at lower doses, with a 50% increase (HR = 1.5, denoted Y) at 43.4 mg and risk doubling (HR = 2.0, denoted Z) at 51.4 mg. Segmented regression identified an inflection point (denoted X) at 63.7 mg (95% CI 62.8−64.7) dexamethasone-equivalent (approximately 425 mg prednisone-equivalent, or 6.1 mg/kg for a 70 kg patient), beyond which the slope increased sixfold (from 0.034 to 0.226 per mg; P < 0.001).

### Exploratory analysis of effect modification

SOFA score at ICU admission significantly modified the dose-response relationship between pre-ICU corticosteroids and IAPA (P for interaction <0.001), whereas BMI, diabetes, and chronic respiratory disease did not (P = 0.163, 0.246, and 0.103, respectively). Stratified dose-response curves are shown in Fig. 4B–E, with corresponding thresholds detailed in Table 2.

**Table 2:** Dose-response thresholds for IAPA risk according to pre-ICU corticosteroid dose and effect modification by patient characteristics.

Subgroup	No. of patients (%)	Inflection point X (95% CI) (mg)^c^	Dose Y for 50% risk increase (mg)	Dose Z for risk doubling (mg)	P for interaction
Overall	2763 (100.0)	63.7 (62.8−64.7)	43.4	51.4	–
BMI^a^					0.163
Underweight	482 (17.4)	64.8 (63.9−65.7)	40.5	48.9	
Normal	1559 (56.4)	62.8 (61.9−63.8)	44.2	52.3	
Overweight/obese	722 (26.13)	64.7 (63.8−65.6)	42.8	50.5	
Diabetes					0.246
Yes	435 (15.7)	63.3 (62.3−64.3)	44.7	52.5	
No	2328 (84.3)	63.4 (62.5−64.3)	43.3	51.4	
Chronic respiratory disease					0.103
Yes	445 (16.1)	69.2 (68.3−70.0)	40.5	46.5	
No	2318 (83.9)	63.2 (62.3−64.2)	43.7	51.8	
SOFA score at admission^b^					<0.001
≤7	2193 (79.4)	59.2 (58.2−60.3)	46	55.1	
>7	570 (20.6)	66.8 (65.8−67.7)	35.2	39.5	

a SOFA score dichotomized at the optimal cut-off value of 7 determined by Youden's index based on the ROC curve for IAPA.

b BMI categories based on World Health Organization (WHO) criteria: underweight (<18.5 kg/m^2^), normal (18.5–24.9 kg/m²), overweight/obese (≥25 kg/m^2^).

c Inflection point X was derived from segmented regression models; Y and Z were obtained by linear interpolation from the RCS dose-response curve; P for interaction was calculated by including a product term between corticosteroid dose (as a continuous variable) and each effect modifier in the IPTW-weighted Cox model with RCS for dose.

Among patients with SOFA > 7, the dose associated with a 50% risk increase (35.2 mg) and risk doubling (39.5 mg) were markedly lower than in those with SOFA ≤ 7 (46.0 mg and 55.1 mg, respectively), indicating heightened susceptibility to corticosteroid-associated IAPA risk in more severely ill patients. In contrast, inflection points were similar across BMI categories (range: 62.8–64.8 mg; overlapping CIs), suggesting comparable risk patterns across underweight, normal-weight, and overweight/obese individuals. Likewise, risk thresholds did not differ meaningfully between diabetic and non-diabetic patients, nor between those with and without chronic respiratory disease.

## Discussion

### Principal findings

In this multicenter cohort study emulating a target trial—the first to our knowledge to quantify the dose-response relationship between pre-ICU corticosteroid exposure and IAPA risk in critically ill patients with influenza—we investigated this association and its clinical implications. Our study yields three key findings. First, we found that any pre-ICU corticosteroid exposure, as a binary variable, is associated with a significantly increased risk of incident IAPA, an effect that persisted after rigorous adjustment for a wide range of confounders. Second, and most importantly, we uncovered a significant nonlinear, J-shaped dose-response relationship. The risk of IAPA began to escalate at cumulative doses well below the threshold for a steep slope change, with a 50% risk increase observed at 43.4 mg and risk doubling at 51.4 mg. A subsequent inflection point at 63.7 mg marked the onset of a dramatically steeper risk increase. Third, we found that this dose-response relationship was significantly modified by disease severity at baseline, with patients presenting with higher SOFA scores (>7) at ICU admission exhibiting markedly lower thresholds for IAPA. These findings advance the management of corticosteroids in severe influenza from a simple binary risk assessment to a dose-aware, actionable framework for clinical decision-making.

### Methodological advantages

Our study offers several methodological advantages. First, by employing counterfactual theory and target trial emulation framework, we were able to balance a comprehensive set of DAG-identified confounders across exposure groups, achieving covariate distributions comparable to those expected under randomization. This approach avoids the "Table 2 Fallacy" inherent in traditional regression analysis [14], and ensures the validity of causal inference [15]. Second, we modeled the dose-response relationship using RCS, which revealed a non-linear, J-shaped association that would have been missed under a linear assumption [16]. Furthermore, by integrating segmented regression, we identified a critical inflection point where the risk begins to escalate steeply, and employed linear interpolation to derive clinically actionable risk thresholds (e.g., doses associated with a 50% and 100% increase in risk). This multi-layered analytical strategy enabled the extraction of actionable evidence from observational data.

### Clinical implications and risk stratification

The high prevalence of pre-ICU corticosteroid use in our cohort (74.2%) is notable, as it reflects a persistent gap between clinical practice and guideline recommendations, which generally discourage the routine use of corticosteroids in influenza due to concerns about secondary infections and increased mortality [[17], [18], [19]]. Our findings provide a nuanced perspective on this issue, moving beyond a simple admonition to avoid corticosteroids to a more precise quantification of the attendant risks. Our observed effect (HR = 2.11) aligns closely with the three recent meta-analyses that have identified pre-ICU corticosteroid use as a significant risk factor for IAPA (pooled effect estimates ranging from 1.89 to 2.43) [2,3,7]. And we further extend this evidence by demonstrating that the risk is not uniform but increases continuously with cumulative dose.

The dose-response thresholds identified in our study have significant clinical implications. Previous studies in influenza populations have suggested that a daily prednisone dose exceeding 0.1 mg/kg [20] or a cumulative dose exceeding 200 mg (approximately 30 mg dexamethasone equivalent) [21] may serve as predictors of IAPA. Our study further refines this risk threshold by precisely quantifying it at 63.7 mg. This inflection point is noteworthy, as it corresponds closely to the cumulative dexamethasone dose commonly recommended in critically ill COVID-19 patients (6 mg/day for 10 days, totaling 60 mg) [[22], [23], [24]]. Remarkably, this threshold exceeds the dose associated with IAPA risk doubling (51.4 mg) in our cohort, suggesting that patients with severe viral pneumonia receiving standard corticosteroid regimens are indeed exposed to a clinically relevant risk of aspergillosis. This concern is corroborated by studies in COVID-19, where a cumulative dose threshold of approximately 60 mg for COVID-19-associated pulmonary aspergillosis (CAPA) has been identified [25,26]. The convergence of these findings across different viral etiologies—influenza and SARS-CoV-2—points to a potential common pathophysiological mechanism: cumulative corticosteroid doses exceeding approximately 60 mg may critically impair host defense against Aspergillus in critically ill, non-neutropenic patients. However, our threshold estimates, while statistically precise, are derived from observational data and should be viewed as exploratory and hypothesis-generating, requiring prospective validation before being adopted as fixed clinical decision points.

Our pre-specified effect modification analysis provides further granularity for IAPA risk stratification. The finding that a higher SOFA score significantly exacerbates the risk per unit of corticosteroid dose is biologically plausible and clinically critical. Patients with profound organ dysfunction and systemic immune dysregulation are inherently more vulnerable [27], and the addition of corticosteroids appears to have a synergistic effect on IAPA risk [28]. In contrast, despite strong biological hypotheses, we did not find significant effect modification by BMI, diabetes, or chronic respiratory disease. Several potential interpretations may explain these null findings. Regarding BMI, the absence of effect modification may reflect the complex and potentially counteracting roles of obesity in critical illness, including its association with the "obesity paradox" [29], which could dilute consistent modifying effect. For diabetes mellitus, the incremental immune impairment attributable to chronic hyperglycemia may become less discernible in the context of overwhelming critical illness and high-dose corticosteroid exposure [30,31]; alternatively, glycemic control during ICU stay (a variable not measured in our cohort) may be more relevant than the pre-existing diagnosis alone [32]. For chronic respiratory disease, the lack of effect modification may reflect the heterogeneous nature of the conditions included (e.g., COPD, asthma) and their potentially divergent impacts on local pulmonary immunity, as well as possible tolerance induced by long-term corticosteroid exposure [33]. Collectively, these analyses move beyond an average population risk estimate, identifying patients with high SOFA scores as particularly vulnerable and providing actionable insights for risk stratification. The lack of significant modification by the other three factors suggests that the heightened risk associated with corticosteroids applies broadly across these subgroups, simplifying the risk stratification model while underscoring the need for vigilance in all critically ill influenza patients receiving corticosteroids.

### Limitations

Our findings should be interpreted within the context of several limitations inherent to observational study designs. First, despite comprehensive adjustment for a wide range of measured confounders identified through DAG, the potential for residual confounding from unmeasured variables remains a consideration. Factors such as the precise indication for corticosteroid therapy (e.g., refractory septic shock vs. reactive airways disease) or the timing of initiation relative to symptom onset could theoretically influence the observed association. More specifically, in the dose-response analysis, residual confounding by indication may persist, as higher corticosteroid doses are often prescribed to patients with more severe underlying disease (e.g., refractory ARDS, septic shock, or severe COPD exacerbations). Although we adjusted for key severity markers (SOFA, CRP, NLR) using RCS and examined effect modification by BMI, diabetes, chronic respiratory disease, and SOFA, we cannot definitively exclude the possibility that the observed dose-response relationship partly reflects differences in the underlying conditions requiring corticosteroid therapy rather than a pure pharmacological dose effect. This is a fundamental limitation of observational dose-response analyses, and our thresholds should be interpreted as exploratory and hypothesis-generating, requiring prospective validation. Second, our exposure definition was intentionally restricted to the 7 days preceding ICU admission to emulate a real-world clinical decision point and to avoid immortal time bias. Consequently, we did not account for the potential impact of cumulative corticosteroid doses administered during the ICU stay, which could further modify IAPA risk—an important aspect warranting further evaluation. Notably, 27.2% of patients in the unexposed group subsequently received corticosteroids at their ICU admission, a post-baseline crossover that would bias our intention-to-treat estimate toward the null, suggesting the reported HR may be conservative. Third, while we applied internationally accepted diagnostic criteria for IAPA, the diagnosis of probable IAPA in critically ill patients remains inherently challenging, and we cannot entirely exclude the possibility of ascertainment bias due to variable clinical awareness. The lower incidence observed in our cohort (6.9%) compared with European cohorts (typically 10–20%) likely reflects differences in diagnostic intensity and geographic epidemiology [1]. Finally, the generalizability of our findings to other geographic regions or healthcare systems may warrant further investigation, as our cohort was derived exclusively from tertiary hospitals in Central China. Differences in patient demographics, circulating influenza strains, and ICU practices may influence the observed thresholds in different settings.

## Conclusions

This study provides the first quantification of the dose-response relationship between pre-ICU corticosteroid exposure and IAPA risk in critically ill, non-neutropenic patients with influenza. The risk is not binary but increases continuously with cumulative dose, with clinically meaningful risk escalation beginning well before a steep inflection point at approximately 64 mg (dexamethasone equivalent). Patients with higher baseline disease severity (SOFA > 7) are particularly vulnerable, exhibiting even lower risk thresholds. Cumulative doses approaching 40−50 mg should prompt a reassessment of the risk-benefit balance and heightened clinical suspicion for IAPA. Our findings move beyond a one-size-fits-all approach toward individualized, dose-aware corticosteroid stewardship—balancing proven benefits against a now-quantified risk of this devastating complication. Future research should validate these thresholds in prospective cohorts and assess whether enhanced surveillance or targeted antifungal prophylaxis benefits patients who cross them.

### CRediT authorship contribution statement

Conceptualization: LP XSN and LHM; Data curation: GLL, QB, LYY, ZYS, HXF, GYY, HB and ZSG; Funding acquisition: LP; Investigation: DH, GLL, QB, LYY, ZYS, HXF, GYY, HB and ZSG; Methodology: LP and BJZ; Project administration: LP and XSN; Resources: XSN, FPL, LJP and ZJF; Writing – original draft: XSN; Writing – review and editing: LP.

## Consent for publication

Not applicable.

## Ethical approval and consent to participate

The study protocol was approved by the ethics committees of all participating hospitals (lead registry number: HNSRY2023-37). Given the non-interventional, observational design and the use of fully anonymized patient data, the requirement for individual informed consent was waived.

## Funding

This work was supported by the Health Commission of Henan Province (Grant No. SBGJ202103021) and the Chinese Preventive Medicine Association (Grant No. HAIC-2024012900107).

## Availability of data and materials

The raw data supporting the conclusions of this article will be made available by the authors, without undue reservation.

## Data availability

The data that has been used is confidential.

Data will be made available on request.

## Declaration of competing interest

The authors declare that they have no competing interests.
