# Supplementary material for: Dose-response relationship of pre-ICU corticosteroids and influenza-associated pulmonary aspergillosis: a multicenter target trial emulation
Source: Ann Intensive Care. 2026 Aug 3;16:100132. doi: 10.1016/j.aicoj.2026.100132 (PMC13476715; doi:10.1016/j.aicoj.2026.100132)
Supplement: Supplementary file 1 [file mmc1.pdf]

Supplementary materials

Supplementary Table S1. Characteristics of pre-ICU corticosteroid exposure among exposed patients 1
Supplementary Table S2. Target trial emulation protocol 2
Supplementary Table S3. The TARGET checklist 3
Supplementary Table S4. Frequency of diagnostic procedures for IAPA, stratified by pre-ICU corticosteroid exposure status 8
Supplementary Table S5. Hypotheses regarding the relationships among measured variables and their causal effects on pre-ICU corticosteroid exposure and IAPA in the DAG framework 9
Supplementary Figure S1. Study timeline illustrating corticosteroid exposure window, ICU admission, and the intention-to-treat framework 62
Supplementary Figure S2. Balance of DAG-derived confounders, stratified by pre-ICU corticosteroid, before and after IPTW 63
Supplementary Figure S3. The mirrored density plot of propensity scores for pre-ICU corticosteroid before and after IPTW 64
Supplementary Figure S4. E-value sensitivity analysis for unmeasured confounders 65

Supplementary Table S1. Characteristics of pre-ICU corticosteroid exposure among exposed patients (n=2050)

Characteristic	n (%)
Documented indication	
ARDS/severe pneumonia	698 (34.0)
COPD/asthma exacerbation	460 (22.4)
Septic shock	399 (19.5)
Autoimmune diseases	158 (7.7)
Other / not documented	335 (16.3)
Corticosteroid molecule	
Methylprednisolone	1103 (53.8)
Dexamethasone	479 (23.4)
Hydrocortisone	266 (13.0)
Prednisone / other	202 (9.9)

Note: Indications were determined based on documented primary reason for initiation or continuation of corticosteroid therapy during the 7-day pre-ICU window, as recorded in electronic health records. The distribution of corticosteroid molecules is based on medication records for exposed patients.

Supplementary Table S2. Target trial emulation protocol

Design element	Target trial protocol	Observational emulation strategy
Eligibility criteria	(1) Age ≥18 years;<br>(2) RT-PCR-confirmed influenza;<br>(3) Admitted to participating ICUs;<br>(4) No neutropenia (ANC ≥ 0.5×10^9/L);<br>(5) No antifungal agents active against *Aspergillus* within 7 days prior to ICU admission	Same as left (see main text and Fig. 1 for details)
Treatment strategies	Strategy A: Initiation or continuation of systemic corticosteroids within 7 days prior to ICU admission;<br>Strategy B: Avoidance of any systemic corticosteroids within 7 days prior to ICU admission	Patients were assigned to Groups A or B based on whether they received systemic corticosteroids during the 7 days prior to ICU admission.<br>The assignment was fixed at ICU admission and followed the ITT principle, i.e., group assignment did not change with post-ICU corticosteroid use
Randomization procedure	Eligible patients were randomized 1∶1 to Strategy A or Strategy B at ICU admission	IPTW was used to emulate randomization and balance baseline covariates across groups
Time zero	ICU admission	ICU admission (at which eligibility assessment, exposure assignment, and follow-up initiation occurred simultaneously)
Follow-up	From time zero to ICU discharge, death, IAPA occurence, or database closure date (whichever occurred first)	Same as left
Outcome	proven or probable IAPA during ICU stay	Same as left

Note: The exposure assessment window (7 days prior to ICU admission) and time zero (ICU admission) are conceptually distinguished. The former is used to retrospectively determine baseline assignment status, while the latter is the common time point for treatment strategy assignment, eligibility confirmation, and follow-up initiation.

Supplementary Table S3. The TARGET (transparent reporting of observational studies emulating a target trial) checklist

Item no.		Checklist item	Location reported
**Abstract**			
**1**	a	Identify that the study attempts to emulate a target trial using observational data. State the study objectives and briefly summarize the specified target trial.	In the section of Background and Methods of Abstract
	b	Report the data sources used for emulation.	In the section of Methods of Abstract
	c	Summarize key assumptions, statistical methods, findings and conclusions.	In Abstract
**Introduction**			
**2**	Background	Describe the scientific background of the study and the gap in knowledge.	In the first paragraph of Introduction
**3**	Causal question	Summarize the causal question.	In the second paragraph of Introduction
**4**	Rationale	Describe the rationale for emulating a target trial with the available data. Cite randomized trials informing the design of the target trial if applicable.	In the second paragraph of Introduction
**Methods**			
**5**	Data sources	Cite the data sources contributing to the analyses and for each one describe the following: original purpose, type, the geographic locations, setting and time-period. If relevant, describe how the data were linked or pooled.	In the section of Study Design

	Target trial specification		Target trial emulation		Location item 6 (specification) reported	Location item 7 (emulation) reported
6	Specify the components of the target trial protocol that would answer the causal question.	7	Describe how the components of the target trial protocol were emulated with the observational data, including how all variables were measured or ascertained.			
	**Eligibility criteria**		**Eligibility criteria**			In the section of Eligibility Criteria
	a	Describe the eligibility criteria.	a	Describe how the eligibility criteria were operationalized with the data.		
	**Treatment strategies**		**Treatment strategies**			In the section of Emulated Intervention Strategies
	b	Describe the treatment strategies that would be compared.	b	Describe how the treatment strategies were operationalized with the data.		
	**Assignment procedures**		**Assignment procedures**			In the section of Emulated Intervention Strategies
	c	Report that eligible individuals would be randomly assigned to treatment strategies and may be aware of their treatment allocation.	c	Describe how assignment to treatment strategies was operationalized with the data.		
	**Follow-up**		**Follow-up**			In the section of Follow-up and Outcomes
	d	Clarify that follow-up would start at time of assignment to the treatment strategies. Specify when follow-up would end.	d	Clarify that follow-up starts at the time individuals were assigned to the treatment strategies. Describe how the end of follow-up was operationalized with the data.		
	**Outcomes**		**Outcomes**			In the section of Follow-up and Outcomes
	e	Describe the outcomes.	e	Describe how the outcomes were operationalized with the data.		

**Causal contrasts**		**Causal contrasts**		
f	Describe the causal contrasts of interest, including effect measures.	f	Describe how the causal contrasts were operationalized with the data, including effect measures.		In the section of Statistical Analysis
**Identifying assumptions**		**Identifying assumptions**		
g	Describe assumptions that would be made to identify each causal estimand. Describe the variables, if any, related to these assumptions.	g.i	For each causal estimand, describe assumptions made to identify it, including assumptions regarding baseline confounding due to lack of randomization.		In the section of Statistical Analysis
		g.ii	Describe how the variables related to these assumptions were operationalized with the data		In the section of Statistical Analysis and in Supplementary Table S2
**Data analysis plan**		**Data analysis plan**		
h	For each causal estimand, describe the data analysis procedures and any associated statistical modelling assumptions, including approaches for handling missing data.	h.i	For each causal estimand, describe the data analysis procedures and any associated statistical modelling assumptions, including approaches for handling missing data.		In the section of Statistical Analysis
		h.ii	For each causal estimand, describe any additional analyses conducted to assess the sensitivity of the results to the choice of operationalizations, assumptions and analysis.		In the section of Statistical Analysis
**Results**				
**8**	Participant selection	Report numbers of individuals assessed for eligibility, eligible, and assigned to each treatment strategy. A flow diagram is strongly recommended.		In the section of Characteristics of Cohort and in Figure 1

**9**	Baseline data	Describe the distribution of characteristics of individuals at baseline, by treatment strategy.	In the section of Characteristics of Cohort and in Table 1
**10**	Follow-up	Summarize length of follow-up and describe reasons for end of follow-up for each treatment strategy and causal contrast.	In the section of Follow-up and Outcomes
**11**	Missing data	Describe the frequency of missing data in all variables, by treatment strategy when applicable.	In the first paragraph from the section of Statistical Analysis and in Table 1
**12**	Outcomes	Describe the frequency or distribution of each outcome, by treatment strategy.	In the section of Characteristics of Cohort
**13**	Effect estimates	Report the effect estimates for each causal contrast with corresponding measures of precision, including both absolute and relative measures of effect, when applicable.	In the third and forth paragraph from the section of Results
**14**	Additional analyses	Report results of all analyses to assess the sensitivity of the estimates to choices in operationalizations, assumptions and analysis.	In the third ,fifth and sixth paragraph from the section of Results
**Discussion**			
**15**	Interpretation	Provide an interpretation of the key findings.	In the section of Discussion
**16**	Limitations	Discuss the limitations of the study considering differences between the target trial and its emulation and the plausibility of assumptions, including assumptions regarding baseline confounding due to lack of randomization.	In the section of Limits
**Other information**			
**17**	Ethics	Provide the institutional research board or ethics committee that approved the study and approval numbers, if relevant.	In the second paragraph from the section of Study Design
**18**	Registration	State whether, when and where the study protocol was registered.	No
**19**	Sharing of	Provide information on whether data, analytic code and/or other materials are accessible, and where and how	In the section of Data availability statement

	study materials	they can be accessed.	
**20**	Funding sources	Provide the sources of funding and detail the role of the funders in the design, conduct and reporting of the study.	In the section of Funding
**21**	Conflicts of interest	State any conflicts of interest and financial disclosures for all authors.	In the section of Conflict of interest

Citation: Cashin AG, Hansford HJ, Hernán MA, Swanson SA, Lee H, Jones MD, et al. Transparent Reporting of Observational Studies Emulating a Target Trial: The TARGET Statement. JAMA. 2025; DOI: 10.1001/jama.2025.13350

Supplementary Table S4. Frequency of diagnostic procedures for IAPA, stratified by pre-ICU corticosteroid exposure status

Diagnostic procedure	Pre-ICU corticosteroid (n=2050)	No pre-ICU corticosteroid (n=713)	χ^2	P value
Bronchoscopy performed, n (%)	1,311 (64.0)	452 (64.0)	0.049	0.825
BAL performed, n (%)	1,229 (60.0)	416 (58.3)	0.502	0.479
Serum galactomannan testing, n (%)	1,484 (72.4)	499 (70.0)	1.393	0.238
BAL fluid galactomannan testing, n (%)	1,007 (49.1)	328 (46.0)	1.938	0.164
Aspergillus PCR, n (%)	625 (30.5)	217 (30.4)	0.000	1.000
Fungal culture, n (%)	1,567 (76.4)	553 (77.6)	0.312	0.577
Chest CT imaging, n (%)	1,858 (90.6)	635 (89.1)	1.313	0.252

Abbreviation: BAL = bronchoalveolar lavage; PCR = polymerase chain reaction; CT = computed tomography.

Note: Diagnostic procedures were performed at the discretion of treating physicians and were not standardized across all participating centers. χ^2 and P values were calculated using Pearson's chi-square test.

Supplementary Table S5. Hypotheses regarding the relationships among measured variables and their causal effects on pre-ICU corticosteroid exposure and IAPA in the DAG framework

Categories	Variables	Group	adjusted variable (yes/no)	DAG paths	Mechanisms by systematic reviews and expert consultations
Demographics	Age	Confounder	Yes	→ Pre-ICU corticosteroid<br>→ IAPA<br>→ Diabetes<br>→ Chronic liver disease<br>→ Chronic kidney disease<br>→ Chronic respiratory disease<br>→ Respiratory rate<br>→ NLR<br>→ CRP<br>→ SOFA<br>→ Bacterial co-infection<br>→ Viral co-infection<br>→ Interleukin-6 inhibitors<br>→ Invasive mechanical ventilation	Age indirectly influences physicians' decision to use corticosteroids prior to ICU admission by affecting the prevalence of underlying diseases (such as asthma, COPD, and other airway diseases), as these underlying conditions are the primary indications for corticosteroid prescribing[1].<br>Immunosenescence impairs the ability of elderly patients to clear influenza virus, and the viral damage to the respiratory mucosa and immune cells (such as macrophages and neutrophils) becomes more severe, thereby greatly increasing the opportunity for secondary aspergillus infection [2].<br>Increasing age is one of the most important risk factors for type 2 diabetes; with advancing age, pancreatic β-cell function declines and insulin resistance increases, leading to a significant rise in the prevalence of diabetes [3].<br>The prevalence and duration of various chronic liver diseases (such as alcoholic liver disease, non-alcoholic fatty liver disease, and cirrhosis) increase with age [4].<br>Renal function naturally declines with age, and the influence of age-related conditions makes chronic kidney disease very common in the elderly population [5].<br>The prevalence of chronic respiratory diseases such as chronic obstructive pulmonary disease and asthma increases with age [6]. Aging leads to decreased elasticity and weakened repair capacity of lung tissue, and the cumulative effects of long-term environmental exposures become apparent [7].<br>Age is an important physiological factor influencing respiratory rate; infants and young children have faster respiratory rates, while the elderly have relatively slower rates, and aging modifies the relationship between blood gas parameters and respiratory rate [8,9].<br>The NLR is significantly higher in the elderly population compared to non-elderly individuals, reflecting age-related alterations in systemic inflammatory status and immunosenescence [10,11].

				→ Time from influenza symptom onset to ICU	Age is an independent predictor influencing the dynamic changes of C-reactive protein (CRP), with elderly patients exhibiting delayed CRP clearance following trauma or inflammatory stimuli [12].<br>In specific disease states such as COVID-19, age demonstrates superior predictive ability for mortality compared to the SOFA score, suggesting a complex relationship between age and the assessment of disease severity [13].<br>Age influences the pathogen distribution patterns in patients with community-acquired pneumonia, with significant differences in bacterial infection detection rates across different age groups [14].<br>Elderly populations exhibit significantly increased susceptibility to respiratory viruses such as influenza virus, and the incidence of viral co-infections is higher in older adults [15].<br>Age is an important factor in clinical decision-making regarding the use of IL-6 inhibitors (such as tocilizumab), with dose adjustments and risk-benefit assessments varying across different age groups [16].<br>Advanced age is a significant risk factor for weaning failure from invasive mechanical ventilation, with elderly patients requiring prolonged weaning at higher rates [17].<br>Elderly patients may experience delayed or atypical recognition of IAPA due to blunted immune responses, nonspecific symptoms, or the presence of multiple comorbidities, leading to variations in the time interval from symptom onset to ICU admission [18,19].<br>**In summary**, age, as a fundamental demographic characteristic of patients with severe influenza, plays a crucial role as an upstream determinant influencing the eight factors described above. Its impact is primarily manifested through three mechanisms: (1) Immunosenescence, leading to increased susceptibility to viral and bacterial infections in the elderly, elevated NLR, and increased risk of co-infections; (2) Altered inflammatory response characteristics, manifested by impaired dynamic clearance of inflammatory markers such as CRP, resulting in acute phase response patterns that differ from younger populations; and (3) Reduced organ functional reserve, making elderly patients more susceptible to respiratory failure requiring invasive mechanical ventilation, with lower weaning success rates. These age-related pathophysiological changes collectively constitute an important basis for the clinical heterogeneity observed in patients with severe influenza, explaining why age should be considered a distal factor influencing multiple clinical variables and outcomes in DAG

					construction.

(Continued)

**Categories**	**Variables**	**Group**	**adjusted variable (yes/no)**	**DAG paths**	**Mechanisms by systematic reviews and expert consultations**

Demographics	BMI	Confounder	Yes	→ Pre-ICU corticosteroid<br>→ IAPA<br>→ Diabetes<br>→ Chronic liver disease<br>→ Chronic kidney disease<br>→ Chronic respiratory disease<br>→ Respiratory rate<br>→ Consolidation<br>→ NLR<br>→ CRP<br>→ SOFA<br>→ Bacterial co-infection<br>→ Interleukin-6 inhibitors<br>→ Invasive mechanical ventilation<br>→ Renal replacement therapy<br>→ ECMO	Clinical studies and practice indicate that obese patients (with high BMI) are more likely to be prescribed systemic corticosteroids when suffering from severe influenza, due to more pronounced inflammatory responses or a higher risk of respiratory complications [20].<br>Low BMI-induced malnutrition leads to impaired immune function, which weakens the body's ability to clear Aspergillus spores[21]. In contrast, high BMI indirectly creates a "breeding ground" for the occurrence of pulmonary aspergillosis by increasing the risk of developing underlying conditions such as COPD[22].<br>Extensive epidemiological and genetic evidence demonstrates that BMI is the most important modifiable risk factor for type 2 diabetes, with a well-established causal association between the two [23].<br>High BMI is a major risk factor for non-alcoholic fatty liver disease[24]. Fatty liver can further progress to hepatitis, liver fibrosis, cirrhosis, and even hepatocellular carcinoma[25,26].<br>Obesity leads to chronic kidney disease[27]. Furthermore, obesity itself can directly cause intraglomerular hypertension and hypertrophy, leading to kidney damage[28].<br>Severe obesity (high BMI) leads to decreased chest wall compliance and impaired respiratory muscle function, causing restrictive ventilatory dysfunction[29]. Low BMI may be associated with inflammatory consumption and malnutrition in obstructive lung diseases (such as COPD)[30], while a high BMI trajectory is linked to accelerated lung function decline and an increased risk of restrictive defects[31].<br>In critically ill patients with influenza, elevated BMI is associated with an increased respiratory rate at ICU admission. Obese patients often exhibit a compensatory increase in respiratory rate due to reduced chest wall compliance and increased ventilatory demand [32].<br>Obesity is a risk factor for an increased extent of pulmonary consolidation on imaging in patients with community-acquired pneumonia and viral pneumonia, possibly due to obesity-related ventilation-perfusion mismatch and abnormal immune responses [32].<br>Obese patients typically have a chronic low-grade inflammatory state, which can affect the peripheral blood NLR, making it elevated at ICU admission. NLR is an important marker of immune response dysregulation in critically ill patients with influenza.<br>Elevated BMI is associated with higher baseline CRP levels. Obesity itself induces systemic inflammation, thereby influencing inflammatory marker levels in critically ill

					patients with influenza at ICU admission [33].<br>Obesity is associated with an increased risk of secondary bacterial pneumonia in critically ill patients with influenza, potentially by altering airway host defense mechanisms and impairing alveolar macrophage function, thereby reducing bacterial clearance [34].<br>Patients with higher BMI who develop severe pneumonia are more likely to receive IL-6 inhibitors (e.g., tocilizumab), as they often present with higher levels of inflammatory markers and more severe systemic inflammation [35].<br>BMI is an independent predictor of the need for invasive mechanical ventilation in critically ill patients with influenza. Obese patients (BMI ≥ 30 kg/m²) are more likely to receive invasive ventilation after ICU admission due to decreased lung compliance and increased respiratory muscle workload [32].<br>Obesity is associated with an increased risk of acute kidney injury (AKI) and the need for renal replacement therapy (RRT) in critically ill patients, possibly mediated by obesity-related inflammation, renal hemodynamic changes, and metabolic factors [36].<br>In patients with severe acute respiratory distress syndrome (ARDS) associated with influenza A (H1N1), obesity (BMI ≥ 30 kg/m²) is an important predictor of the need for extracorporeal membrane oxygenation (ECMO) therapy, reflecting the higher risk of poor response to conventional mechanical ventilation support in obese patients [37,38].<br>**In summary**, BMI is directly associated with corticosteroid use: patients with high BMI are more likely to receive systemic corticosteroids prior to ICU admission due to more pronounced inflammatory responses or a higher risk of respiratory complications. At the same time, BMI is a key determinant of various comorbidities, all of which may increase the risk of IAPA through distinct mechanisms or influence clinical decision-making. In terms of clinical features at ICU admission, high BMI is closely associated with increased respiratory rate, greater extent of pulmonary consolidation, elevated levels of inflammatory markers, and an increased risk of secondary bacterial pneumonia. Furthermore, BMI significantly affects interventions at ICU admission.

(Continued)

Categories	Variables	Group	adjusted variable (yes/no)	DAG paths	Mechanisms by systematic reviews and expert consultations
Demographics	Smoking	Confounder	Yes	→ Pre-ICU corticosteroid<br>→ IAPA<br>→ Diabetes<br>→ Chronic liver disease<br>→ Chronic kidney disease<br>→ Chronic respiratory disease<br>→ Respiratory rate<br>→ Consolidation<br>→ NLR<br>→ CRP<br>→ SOFA<br>→ Bacterial co-infection<br>→ Viral co-infection<br>→ Invasive mechanical ventilation	Smoking is associated with an increased likelihood of corticosteroid prescription prior to ICU admission, as smokers with influenza often present with more severe pulmonary inflammation and underlying chronic respiratory conditions that clinicians consider when initiating anti-inflammatory therapy.<br>The impact of smoking on the development of pulmonary aspergillosis primarily occurs through three aspects. (1)Direct Damage: It impairs respiratory ciliary clearance function and the epithelial barrier, increasing the risk of fungal inhalation and colonization [39]. (2)Creating Underlying Diseases: Smoking leads to chronic lung diseases such as COPD, which are established high-risk factors for chronic pulmonary aspergillosis [40]. (3)Influencing Treatment: The use of high-dose inhaled corticosteroids to control COPD symptoms further increases the risk of fungal colonization [41,42].<br>Smoking is an important risk factor for type 2 diabetes. It significantly increases the risk of new-onset diabetes through mechanisms such as enhancing insulin resistance, promoting chronic inflammation, inducing oxidative stress, and directly impairing pancreatic β-cell function [43].<br>Studies indicate that smoking is a risk factor for chronic liver disease [44]. Toxic substances in tobacco may exacerbate liver injury through mechanisms such as inducing oxidative stress and inflammatory responses [45,46].<br>Smoking is a preventable cause of chronic kidney disease (CKD). It increases the risk of new-onset CKD and accelerates the progression of existing CKD to end-stage renal disease [47]. Additionally, inhaled cadmium (Cd) may be one of the biological pathways through which smoking contributes to renal function impairment [48].<br>The relationship between smoking and chronic respiratory disease is one of the most classic and well-established causal links. Long-term smoking is the primary cause of chronic obstructive pulmonary disease (COPD), emphysema, chronic bronchitis, and other chronic respiratory system diseases [49].<br>Smoking increases respiratory effort by enhancing pulmonary inflammation and airway

					resistance. Animal studies have shown that mice exposed to cigarette smoke exhibit more severe acute respiratory dysfunction following influenza virus infection, manifested as a significantly increased respiratory rate [50].<br>Smoking exacerbates influenza virus-induced pulmonary pathological damage. Research has found that cigarette smoke-exposed mice infected with influenza virus develop more extensive pulmonary inflammatory infiltration, alveolar microvascular occlusion, and vascular leakage, which manifest radiologically as an increased extent of pulmonary consolidation [50].<br>Smoking promotes neutrophil recruitment to the lungs and suppresses lymphocyte function. Studies have confirmed that in cigarette smoke-exposed mice with influenza virus infection, pulmonary recruitment of neutrophils, monocytes, and macrophages is significantly increased, while alveolar macrophages are depleted. This immune cell imbalance may be reflected peripherally as an elevated NLR [51].<br>Smoking enhances the inflammatory response induced by viral infection. Research has shown that cigarette smoke-exposed mice infected with influenza virus exhibit significantly elevated levels of pro-inflammatory cytokines, such as interleukin-6 (IL-6), in the lungs. C-reactive protein (CRP), an acute-phase protein produced by the liver in response to IL-6, is correspondingly increased [52].<br>Smoking is associated with a higher risk of organ dysfunction in patients with severe influenza,which may contribute to more severe multi-organ dysfunction, reflected in higher Sequential Organ Failure Assessment (SOFA) scores [53].<br>Smoking is an independent risk factor for community-acquired pneumonia. A meta-analysis demonstrated that smokers have a 1.88- to 4.1-fold higher risk of pneumococcal pneumonia compared to non-smokers [54]. Furthermore, studies have confirmed that influenza virus infection can enhance bacterial proliferation in the airways of smokers, making them more susceptible to viral-bacterial co-infections [55].<br>Smoking is associated with an increased risk of influenza virus infection. A case-control study of kidney transplant recipients found that active smokers had a 13.1-fold higher risk of laboratory-confirmed influenza infection compared to non-smokers (aOR 13.1, 95% CI 2.3–76) [56]. Additionally, a Mendelian randomization study showed that genetic susceptibility to smoking was associated with an increased risk of influenza (OR 1.70, 95%

					CI 1.09–2.65) [57].<br>Smoking is associated with an increased risk of requiring mechanical ventilation in patients with influenza. A large-scale study of hospitalized influenza patients demonstrated that individuals who use substances (with high smoking rates) had a significantly higher risk of requiring mechanical ventilation compared to non-users [58]. Mechanistically, smoking-induced pulmonary microvascular occlusion and impaired gas exchange increase the need for respiratory support [59].<br>**In summary**, the mechanisms of smoking primarily operate through three dimensions: first, direct lung injury. Smoking impairs respiratory ciliary clearance and the epithelial barrier, increasing the risk of fungal inhalation and colonization; at the same time, by enhancing pulmonary inflammation and airway resistance, it leads to a significant increase in respiratory rate following influenza virus infection and exacerbates the extent of pulmonary consolidation. Second, induction of underlying diseases. Smoking is the primary cause of chronic respiratory diseases (e.g., COPD) and is an independent risk factor for type 2 diabetes, chronic liver disease, and chronic kidney disease. These underlying conditions themselves may influence the decision to prescribe corticosteroids and the risk of developing IAPA. Third, regulation of immune and inflammatory responses. Smoking promotes neutrophil recruitment to the lungs and suppresses lymphocyte function, resulting in an elevated NLR; it also enhances virus-induced inflammatory responses, leading to increased levels of inflammatory markers such as IL-6 and CRP. This immune imbalance and systemic inflammatory response further aggravate organ dysfunction, manifesting as higher SOFA scores and increasing the need for mechanical ventilation in influenza patients. In addition, smoking significantly increases the risk of influenza virus infection and bacterial co-infection. In summary, within the DAG, smoking points to the aforementioned clinical variables through three pathways: direct injury, induction of underlying diseases, and regulation of immune-inflammatory responses.

(Continued)

Categories	Variables	Group	adjusted variable (yes/no)	DAG paths	Mechanisms by systematic reviews and expert consultations
Comorbidities	Diabetes	Confounder	Yes	→ Pre-ICU corticosteroid<br>→ IAPA<br>→ Hemoptysis<br>→ NLR<br>→ CRP<br>→ SOFA<br>→ Bacterial co-infection<br>→ Invasive mechanical ventilation<br>→ Renal replacement therapy<br>→ ECMO	Patients with diabetes, particularly those with poor glycemic control, are more prone to severe inflammatory responses and acute respiratory distress syndrome after influenza infection, leading clinicians to be more inclined to initiate corticosteroid therapy prior to ICU admission to control excessive inflammation [60].<br>Diabetes is an independent risk factor for invasive pulmonary aspergillosis. Studies have shown that even as the sole risk factor, diabetic patients have a 27% higher risk of developing IPA compared to the general population, a mechanism potentially related to diabetes-associated immune dysfunction [61].<br>Hemoptysis is a common clinical manifestation of pulmonary aspergillosis. In clinical studies analyzing the characteristics of patients with hemoptysis, diabetic patients account for a certain proportion (33.3% in the severe hemoptysis group), suggesting that diabetes may be an underlying disease contributing to hemoptysis [62].<br>The neutrophil-to-lymphocyte ratio is an indicator reflecting inflammatory and immune status. Research indicates that diabetes significantly affects NLR, suggesting an alteration in the inflammatory-immune balance in diabetic patients [63].<br>Diabetes is closely associated with systemic inflammatory responses. Studies have found that C-reactive protein levels are significantly elevated in diabetic patients, and there is a strong correlation between HbA1c levels and CRP, indicating that hyperglycemia exacerbates the inflammatory response [64].<br>Diabetes exacerbates organ dysfunction in critically ill patients. Evidence confirms that in patients with sepsis, diabetes significantly worsens SOFA scores, indicating more severe organ failure in diabetic individuals [65].<br>Diabetic patients have an increased susceptibility to bacterial infections. Research indicates that diabetic patients are more prone to secondary bacterial infections following viral infections, possibly related to impaired immune function and a hyperglycemic environment conducive to bacterial growth [66].<br>Diabetes is a risk factor for the need for invasive mechanical ventilation in critically ill

					patients. In neurocritical patients, glycemic control indicators (such as admission blood glucose gap and HbA1c) are significantly associated with the risk of requiring invasive mechanical ventilation [67].<br>Diabetes is a leading cause of chronic kidney disease and an important risk factor for acute kidney injury in ICU patients. Diabetic patients are more prone to severe renal impairment requiring renal replacement therapy during critical illness [68].<br>When diabetic patients develop severe influenza, the disease progresses more rapidly, and they are more likely to develop respiratory or circulatory failure that is difficult to correct with conventional treatment, potentially necessitating ECMO support [60].<br>**In summary**, based on the available evidence, diabetes exerts influence through multiple pathways in the clinical course of critically ill influenza patients. First, at the level of treatment decisions, diabetic patients are more susceptible to severe inflammatory responses and acute respiratory distress syndrome after influenza infection, which often prompts clinicians to initiate corticosteroid therapy prior to ICU admission to control excessive immune inflammation. Secondly, in terms of disease susceptibility, diabetes itself is an independent risk factor for invasive pulmonary aspergillosis; the immune dysfunction it causes significantly increases patients' baseline risk of developing IAPA, while also increasing the likelihood of secondary bacterial infections. Third, regarding disease severity and clinical manifestations, diabetes is not only associated with IAPA-related clinical presentations such as hemoptysis, but also exacerbates systemic inflammatory responses (e.g., elevated CRP, altered NLR) and worsens organ dysfunction (e.g., increased SOFA scores), thereby increasing the patient's need for advanced life support measures (such as invasive mechanical ventilation, renal replacement therapy, and even ECMO).

(Continued)

Categories	Variables	Group	adjusted variable (yes/no)	DAG paths	Mechanisms by systematic reviews and expert consultations
Comorbidities	Chronic liver disease	Confounder	Yes	→ Pre-ICU corticosteroid<br>→ IAPA<br>→ Hemoptysis<br>→ Respiratory rate<br>→ Consolidation<br>→ SOFA<br>→ Bacterial co-infection<br>→ Invasive mechanical ventilation<br>→ Renal replacement therapy<br>→ ECMO<br>→ Time from influenza symptom onset to ICU<br>→ Admission source<br>→ Antifungal prophylaxis<br>→	Corticosteroids have therapeutic applications during acute exacerbations of CLD [69], such as in acute-on-chronic liver failure (ACLF) [70].<br>CLD is a well-established risk factor for pulmonary aspergillosis [71], as underlying conditions (including chronic liver and kidney diseases) elevate the infection risk [72].<br>Reduced synthesis of coagulation factors and abnormal coagulation function due to CLD can lead to respiratory tract bleeding, manifesting as hemoptysis, when combined with pulmonary diseases such as influenza [73].<br>CLD patients are at high risk for severe influenza, which often presents with rapid respiratory rate and dyspnea [74], and may show consolidation on pulmonary imaging [75].<br>The SOFA score is specifically designed to assess the degree of organ failure in patients with chronic liver disease [76].<br>Patients with liver cirrhosis have hepatic dysfunction-associated immune impairment, including impaired neutrophil function and reduced chemotactic capacity, which significantly increases the risk of bacterial co-infections such as bacterial sepsis following influenza infection [77].<br>CLD patients are at higher risk for severe influenza, which can progress to respiratory failure requiring invasive mechanical ventilation [78]. Additionally, they may develop hepatorenal syndrome or acute kidney injury, necessitating renal replacement therapy upon meeting dialysis criteria [79], and may require ECMO support in cases of severe ARDS [80].<br>The immunocompromised status of CLD patients may lead to more rapid progression of influenza, potentially shortening the time from symptom onset to ICU admission [81,82].<br>Patients with chronic liver disease, particularly those with cirrhosis, are more susceptible to hepatic decompensation (manifested as ascites, hepatic encephalopathy, etc.) following influenza infection due to their underlying liver condition, leading to increased hospitalization requirements and a higher likelihood of ICU admission [83].<br>Chronic liver disease (CLD) patients have compromised immune function, significantly increasing their risk of invasive fungal infections. Clinically, antifungal prophylaxis may be

				Broad-spectrum antibiotic	administered based on the degree of immunodeficiency caused by liver impairment [84]. Additionally, it should be noted that in severe influenza patients with CLD, due to impaired drug metabolism and excretion caused by liver dysfunction, the risk of toxic reactions (such as hepatotoxicity) from using antifungal drugs for infection prophylaxis is significantly increased [85]. Therefore, when the indications for prophylactic antifungal use are carefully assessed, the usage rate may be reduced. In contrast, patients without such underlying conditions have better drug tolerance, resulting in a more favorable benefit-risk ratio for prophylactic use, which may lead to a relatively higher usage rate.<br>Due to decreased immunity, CLD patients are prone to co-infections caused by multiple resistant bacterias when sever influenza occurs, leading to the empirical use of broad-spectrum antibiotics [86].<br>**In summary**, CLD can directly or indirectly influence the clinical course and medical decisions for influenza patients through various pathophysiological mechanisms. Direct mechanisms primarily include: (1)immune suppression, (2)coagulation dysfunction, and (3)organ insufficiency. Indirect mechanisms are reflected in the fact that CLD is a recognized high-risk factor for severe influenza. Patients are more prone to rapid progression to severe disease, presenting with symptoms such as high respiratory rate and pulmonary consolidation, thereby increasing the likelihood of requiring invasive mechanical ventilation and ECMO. The accelerated disease progression may also shorten the time from symptom onset to ICU admission.

(Continued)

Categories	Variables	Group	adjusted variable (yes/no)	DAG paths	Mechanisms by systematic reviews and expert consultations
Comorbidities	Chronic kidney disease	Confounder	Yes	→ Pre-ICU corticosteroid<br>→ IAPA<br>→ Hemoptysis<br>→ NLR<br>→ CRP<br>→ SOFA<br>→ Bacterial co-infection<br>→ Interleukin-6 inhibitors<br>→ Invasive mechanical ventilation<br>→ Renal replacement therapy<br>→ ECMO<br>→ Time from influenza symptom onset to ICU<br>→ Admission source<br>→ Antifungal prophylaxis	Patients with Chronic kidney disease (CKD) may be more likely to have received corticosteroid treatment before ICU transfer due to comorbid conditions (such as chronic obstructive pulmonary disease or autoimmune diseases) or the risk of adrenal insufficiency [87,88].<br>Patients with CKD often have platelet dysfunction and vascular endothelial lesions, which increase the risk of hemoptysis during severe respiratory infections or invasive fungal infections [89].<br>In patients with CKD, a persistent state of microinflammation and immune dysregulation is present. This state drives aberrant activation of the innate immune system (represented by neutrophils) while suppressing adaptive immunity (represented by lymphocytes), leading to a significant elevation in the neutrophil-to-lymphocyte ratio (NLR). A meta-analysis published in 2025 indicated that a high NLR is significantly associated with adverse outcomes in CKD patients, confirming the value of NLR as a marker of CKD-related systemic inflammation and immune dysregulation [90].<br>In patients with CKD, particularly after a marked decline in glomerular filtration rate or upon progression to end-stage renal disease (ESRD), C-reactive protein (CRP) levels are typically higher than those in healthy individuals, irrespective of the presence or absence of concurrent infection [91,92].<br>The renal component of the Sequential Organ Failure Assessment (SOFA) score is directly determined by creatinine or urine output. Since patients with CKD have higher baseline creatinine levels, their admission SOFA score will be higher. CKD is the cause of the higher SOFA score upon ICU admission [76].<br>CKD impairs both acquired and innate immune function, a state referred to as "immunological fragility" [93]. Multiple studies have confirmed that patients with CKD have a significantly increased susceptibility to bacterial infections. A review on infectious complications in CKD explicitly noted that the hospitalization rate for bacterial infections such as pneumonia and bacteremia/sepsis is three to four times higher in patients with CKD

				→<br>Broad-spectrum antibiotic	compared with the general population [94]. Recent studies have also emphasized that dysregulation of the complement system in CKD patients represents one of the key pathogenic mechanisms underlying this increased infection risk [95].<br>CKD itself is a chronic inflammatory state, characterized by elevated cytokines such as interleukin-6 [96]. During viral pneumonia-induced cytokine storms, patients with CKD may be considered at higher risk for severe inflammatory responses and potentially benefit from interleukin-6 inhibitors, influencing treatment decisions [97].<br>Patients with CKD have poor cardiopulmonary reserve and reduced compensatory capacity for acute lung injury, making them more likely to develop respiratory failure requiring invasive mechanical ventilation [98].<br>Influenza infection can induce or exacerbate acute kidney injury (AKI) [99]. On top of pre-existing CKD, this makes it easier to reach the threshold for requiring renal replacement therapy [100].<br>CKD is a key consideration in evaluating patients with severe respiratory or circulatory failure for extracorporeal membrane oxygenation (ECMO). Although severe CKD may be a relative contraindication for ECMO, in cases of refractory shock or respiratory failure, the presence of CKD itself is an upstream factor influencing the decision to initiate ECMO [101,102].<br>Due to weakened immune clearance and reduced organ reserve, patients with CKD may progress more rapidly to severe illness after influenza infection, thereby shortening the time from symptom onset to ICU admission [103].<br>Due to their poorer baseline health, patients with CKD are more likely to be transferred earlier and more frequently from the emergency department or general wards to the ICU during influenza illness. In other words, the CKD status of influenza patients affects the pathway and source of ICU admission [103].<br>CKD, especially end-stage renal disease (ESRD), leads to immunosuppression (such as uremia-related immune dysfunction), which is a known independent risk factor for invasive pulmonary aspergillosis [104,105]. Additionally, As described in the preceding section regarding the factors of chronic liver diseases,it should be noted that in severe influenza patients with CKD, due to impaired drug metabolism and excretion caused by kidney dysfunction, the risk of toxic reactions (such as nephrotoxicity) from using antifungal drugs

					for infection prophylaxis is significantly increased [85]. Therefore, when the indications for prophylactic antifungal use are carefully assessed, the usage rate may be reduced. In contrast, patients without such underlying conditions have better drug tolerance, resulting in a more favorable benefit-risk ratio for prophylactic use, which may lead to a relatively higher usage rate.<br>In patients with CKD, viral pneumonia is more likely to progress rapidly [106], and secondary bacterial pathogens may be more complex (such as multidrug-resistant Gram-negative bacteria) [106]. Additionally, since the dosing of some antibiotics depends on renal function, clinicians may be more inclined to use broad-spectrum antibiotics [107].<br>**In summary**, CKD can serve as an upstream factor for the above elements in this DAG, primarily based on its dual role as an immunosuppressed state and a foundation of organ dysfunction. Meanwhile, CKD influences baseline disease severity (e.g., higher SOFA scores), accelerates disease progression (shortening the time to ICU admission), and, due to its common comorbid status, affects pre-ICU corticosteroid use and the source of ICU admission.

(Continued)

**Categories**	**Variables**	**Group**	**adjusted variable (yes/no)**	**DAG paths**	**Mechanisms by systematic reviews and expert consultations**
Comorbidities	Chronic respiratory disease	Confounder	Yes	→ Pre-ICU corticosteroid<br>→ IAPA<br>→ Hemoptysis<br>→ Respiratory rate<br>→ Consolidation<br>→ NLR<br>→ CRP<br>→ SOFA<br>→ Bacterial co-infection<br>→ Viral co-infection<br>→ Interleukin-6 inhibitors<br>→ Invasive mechanical ventilation<br>→ Renal replacement therapy<br>→ ECMO<br>→ Time from influenza	Chronic Respiratory Disease (CRD) patients (e.g., with asthma, COPD) may use corticosteroids chronically or during acute phases [108].<br>CRD causes structural lung damage (such as bronchiectasis, pulmonary fibrosis) and impairs immune barriers, creating a high-risk foundation for fungal (especially Aspergillus) colonization and infection [109].<br>CRD is often associated with fragile, proliferative bronchial blood vessels, which are prone to rupture and bleeding during intense coughing or secondary infections triggered by influenza [110].<br>CRD leads to poor pulmonary reserve. viral pneumonia can rapidly worsen airway obstruction and ventilation/perfusion mismatch, triggering compensatory tachypnea [111].<br>Patients with CRD have impaired airway clearance, making them susceptible to severe secondary bacterial (e.g., Staphylococcus aureus, Streptococcus pneumoniae) or fungal infections, leading to pulmonary consolidation [112].<br>In patients with chronic respiratory diseases, the NLRP3 inflammasome is in a pre-activated state, which can be further upregulated following influenza virus infection, thereby influencing the levels of inflammatory markers such as NLR [113,114].<br>In patients with chronic respiratory diseases (e.g., COPD), influenza virus infection can activate the NLRP3 inflammasome pathway, promoting the secretion of pro-inflammatory cytokines such as IL-1β and IL-18, which in turn affect the levels of systemic inflammatory markers like CRP [115].<br>The fragile respiratory reserve in CRD patients rapidly decompensates under severe pneumonia and Acute Respiratory Distress Syndrome (ARDS) induced by influenza, and is prone to inducing or exacerbating systemic inflammatory response and multiple organ dysfunction, thereby elevating the Sequential Organ Failure Assessment (SOFA) score at ICU admission [116].<br>In patients with chronic obstructive pulmonary disease, there is a synergistic relationship between influenza virus infection and bacterial co-infection—influenza virus can enhance the

				symptom onset to ICU → Antifungal prophylaxis → Broad-spectrum antibiotic	proliferation of bacteria (e.g., non-typeable Haemophilus influenzae) in the airways, exacerbating inflammatory lung injury [117]. Additionally, viral respiratory infections in patients with chronic respiratory diseases are significantly associated with colonization and infection by pathogenic bacteria such as Streptococcus pneumoniae and Haemophilus influenzae [118,119].<br>Patients with chronic respiratory diseases have a higher rate of inhaled corticosteroid use, which significantly increases their susceptibility to non-influenza respiratory viruses (e.g., adenovirus, respiratory syncytial virus) [120]. Moreover, patients with chronic underlying conditions and compromised immune function are more prone to co-infection with multiple respiratory viruses [121].<br>CRD patients are prone to more intense inflammatory responses (cytokine storm) after influenza infection, which becomes a consideration for clinical decisions to use immunomodulators (e.g., tocilizumab) [122].<br>CRD patients have very low pulmonary reserve [123]. Once severe respiratory failure occurs post-respiratory viral infections, they respond poorly to non-invasive ventilation, significantly increasing the probability of requiring invasive mechanical ventilation [124].<br>Severe hypoxemia and septic shock can lead to acute kidney injury [125]. CRD, as a foundation for severe disease progression, increases the risk of requiring renal replacement therapy [126].<br>When CRD patients develop refractory respiratory or circulatory failure due to influenza, their underlying condition is one of the factors considered in deciding to initiate Extracorporeal Membrane Oxygenation (ECMO) as an ultimate support strategy [127].<br>As mentioned above, CRD patients have very low pulmonary reserve [123]. The influenza virus can trigger severe respiratory inflammation, epithelial damage, and their acute exacerbations, thereby shortening the time from symptom onset to ICU admission [116].<br>CRD causes structural lung damage (such as bronchiectasis, pulmonary fibrosis) and impairs immune barriers, creating a high-risk foundation for fungal (especially Aspergillus) colonization and infection [109], and consequently serves as a basis for clinical decisions to initiate prophylactic antifungal therapy [129].<br>Given the high risk of secondary infection and potentially complex pathogens in CRD patients, clinicians are more inclined to empirically initiate broad-spectrum antibiotics to

					cover possible resistant bacteria [108].<br>**In summary**, when drawing the DAG, arrows from CRD should primarily point to the following two categories of factors: (1) Direct complications. Such as pulmonary aspergillosis, hemoptysis, high respiratory rate, and pulmonary consolidation. These are direct or predisposed outcomes of chronic lung pathological changes under the stress of acute infection. (2) Specific clinical interventions. Such as broad-spectrum antibiotics, antifungal prophylaxis, interleukin-6 inhibitors, invasive mechanical ventilation, ECMO, CRRT, and pre-ICU corticosteroids. In these cases, the presence of CRD directly alters the threshold and pathway for clinical decision-making and treatment, serving as a crucial basis for physicians to adopt these interventions.

(Continued)

Categories	Variables	Group	adjusted variable (yes/no)	DAG paths	Mechanisms by systematic reviews and expert consultations
Clinical features at adimission	Hemoptysis	Confounder	Yes	→ Pre-ICU corticosteroid<br>→ IAPA<br>→ Interleukin-6 inhibitors<br>→ Invasive mechanical ventilation<br>→ ECMO<br>→ Antifungal prophylaxis	Hemoptysis at ICU admission may prompt clinicians to initiate systemic corticosteroids prior to ICU admission, as hemoptysis in severe influenza is often associated with diffuse alveolar hemorrhage or underlying pulmonary aspergillosis, conditions for which corticosteroids are frequently prescribed despite limited evidence [130,131].<br>For patients with severe viral pneumonia including influenza, severe lung tissue damage and immunosuppression can lead to hemoptysis. [132,133]. The presence of blood and necrotic tissue provides a growth medium for inhaled Aspergillus[134], allowing it to colonize damaged lung tissue and vascular injuries, thereby increasing the risk of invasive fungal infections (such as pulmonary aspergillosis) [135,136].<br>Hemoptysis reflects an excessive inflammatory response in patients with severe viral pneumonia (e.g., cytokine storm), leading to the use of IL-6 inhibitors for inflammation control [133].<br>It can also cause airway obstruction, bleeding, or respiratory failure [137], necessitating invasive mechanical ventilation support [137]. Furthermore, severe hemoptysis accompanied by refractory respiratory failure or acute respiratory distress syndrome (ARDS) may require ECMO as salvage therapy [138].<br>The presence of blood and necrotic tissue in patients with severe viral pneumonia provides a growth medium for inhaled Aspergillus[134], allowing it to colonize damaged lung tissue and vascular injuries, thereby increasing the risk of invasive fungal infections (such as pulmonary aspergillosis) and prompting clinicians to initiate antifungal prophylaxis [135,136].<br>**In summary**, in severe influenza patients, hemoptysis serves both as a marker of lung tissue damage and an increased risk of secondary invasive fungal infections, as well as a trigger for initiating corticosteroids. Simultaneously, the excessive inflammation indicated by hemoptysis and the resulting respiratory failure often drive the escalation of clinical treatment, including the use of IL-6 inhibitors, invasive mechanical ventilation, and even ECMO support.

(Continued)

Categories	Variables	Group	adjusted variable (yes/no)	DAG paths	Mechanisms by systematic reviews and expert consultations
Clinical features at adimission	Respiratory rate > 30/min	Confounder	Yes	→ Pre-ICU corticosteroid<br>→ IAPA<br>→ Interleukin-6 inhibitors<br>→ Invasive mechanical ventilation<br>→ Renal replacement therapy<br>→ ECMO<br>→ Antifungal prophylaxis<br>→ Broad-spectrum antibiotic	Respiratory rate > 30/min at ICU admission, indicative of acute respiratory distress or severe respiratory compromise, has been identified as a key clinical driver for the initiation of systemic corticosteroids prior to ICU admission in patients with severe influenza [139,140].<br>Respiratory rate >30/min is an important clinical indicator of tachypnea, which is an early sign of severe influenza and respiratory failure [141,142], and such patients are at high risk for invasive pulmonary aspergillosis [143].<br>Severe tachypnea in viral penumonia patients may indicate an excessive systemic inflammatory response or "cytokine storm," which is an indication for immunomodulatory therapies such as interleukin-6 inhibitors [145, 146]. Consequently, tachypnea can serve as a decision node for initiating such treatments.<br>When tachypnea progresses to severe respiratory distress and hypoxemia in critically ill patients with viral pneumonia, i.e., respiratory failure, invasive mechanical ventilation becomes the core life-support measure. Tachypnea is an important clinical indicator for deciding whether intubation and mechanical ventilation are required [147]. Additionally, severe influenza can lead to multiple organ dysfunction syndrome, including acute kidney injury. When tachypnea is accompanied by oliguria, fluid overload, and other manifestations, renal replacement therapy may be necessary [148].<br>Furthermore, for refractory hypoxemia that is difficult to correct with invasive mechanical ventilation (i.e., refractory respiratory failure), tachypnea is part of its clinical presentation. In such cases, ECMO is the ultimate life-support option [149,150].<br>Respiratory rate >30/min is an important clinical indicator of tachypnea, which is an early sign of severe influenza and respiratory failure [141,142], and such patients are at high risk for invasive pulmonary aspergillosis [143]. Therefore, the onset of tachypnea may prompt clinicians to evaluate and initiate prophylactic antifungal therapy.<br>In clinical practice, when managing critically ill influenza patients with tachypnea, especially when the pathogen is unknown or a bacterial co-infection is suspected, the

					empirical use of broad-spectrum antibiotics is a common therapeutic decision [144].<br>**In summary**, the reason tachypnea can point to these seemingly different clinical interventions or outcomes is that it is not an isolated symptom but rather a common external manifestation of a series of severe pathophysiological changes within patients with severe influenza. It signifies that the influenza has progressed to a critical state involving severe lower respiratory tract inflammation, hypoxemia, and even multiple organ involvement. The logical pathway is as follows: Tachypnea, acting as a sensitive clinical marker for respiratory failure and systemic hyperinflammatory response, directly triggers advanced life support (such as mechanical ventilation and ECMO) and the prevention and treatment of severe complications (such as antifungal prophylaxis). Simultaneously, it also serves as a basis for deciding to initiate empirical broad-spectrum antibiotics and immunomodulatory therapy. Ultimately, when multiple organ functions are affected (e.g., concurrent acute kidney injury), tachypnea becomes part of the clinical context indicating the need for comprehensive organ support, such as renal replacement therapy.

(Continued)

Categories	Variables	Group	adjusted variable (yes/no)	DAG paths	Mechanisms by systematic reviews and expert consultations
Clinical features at adimission	Consolidation	Confounder	Yes	→ Pre-ICU corticosteroid<br>→ IAPA<br>→ Interleukin-6 inhibitors<br>→ Invasive mechanical ventilation<br>→ Renal replacement therapy<br>→ ECMO<br>→ Antifungal prophylaxis<br>→ Broad-spectrum antibiotic	Pulmonary consolidation is an important decision-making basis for clinicians to initiate corticosteroid therapy in critically ill influenza patients, as it reflects severe pulmonary inflammatory response and acute lung injury, prompting clinicians to use corticosteroids to suppress excessive immune response [151].<br>Severe influenza patients with extensive lung consolidation are at high risk for influenza-associated invasive pulmonary aspergillosis[152]. Influenza virus itself can suppress the recruitment of neutrophils in the lungs [153], disrupt local immune defenses, and thereby increase the risk of secondary invasive pulmonary aspergillosis [154]. Extensive lung consolidation is precisely the imaging manifestation of such severe lung injury and immune dysregulation [155].<br>Severe influenza pneumonia can trigger an excessive host inflammatory response (cytokine storm), in which IL-6 is a key pro-inflammatory factor [156]. Although large-scale RCT evidence is lacking for influenza, immune modulators targeting pathways such as IL-6 have been discussed in systematic reviews as potential therapeutic strategies. Their application is premised on severe inflammatory lung injury, such as extensive consolidation [157].<br>Diffuse lung consolidation often leads to severe hypoxemia and respiratory failure, which is a primary indication for invasive mechanical ventilation [158].<br>Severe influenza can cause multiple organ dysfunction, including acute kidney injury (due to factors such as rhabdomyolysis or shock). Renal replacement therapy (such as continuous renal replacement therapy, CRRT) is an important component of supportive care. The systemic severe inflammation and hypoxic state represented by lung consolidation are among the driving factors leading to such distant organ injury [159,160]. When influenza pneumonia causes severe respiratory failure and oxygenation cannot be maintained even with invasive mechanical ventilation, extracorporeal membrane oxygenation (ECMO) can serve as a salvage therapy.<br>Severe influenza patients with extensive lung consolidation are at high risk for

					influenza-associated invasive pulmonary aspergillosis (IAPA). Clinical studies have explored whether prophylactic antifungal therapy (such as posaconazole) in these patients can reduce the incidence of IAPA, reflecting the guiding role of consolidation as a high-risk marker for clinical preventive decisions [152].<br>Lung consolidation caused by severe influenza is often clinically difficult to distinguish from secondary bacterial pneumonia (especially drug-resistant infections), and patients are in an immunosuppressed state with a high risk of infection. Therefore, empirical use of broad-spectrum antibiotics is necessary to cover common and resistant pathogens to reduce mortality [161-163].<br>**In summary**, in the causal pathway diagram for critically ill influenza patients, imaging findings of lung consolidation serve as a key clinical node that can directly point to multiple downstream treatments. The logic is as follows: lung consolidation is a visual marker of severe lung injury and inflammatory response, (1) directly driving the need for advanced respiratory support (invasive mechanical ventilation, ECMO); (2) indicating a significantly increased risk of secondary bacterial or fungal infections, thereby guiding the use of broad-spectrum antibiotics and antifungal prophylaxis; (3) reflecting a systemic hyperinflammatory state, providing a theoretical basis for the use of immune modulators such as IL-6 inhibitors; (4) as a local manifestation of a systemic disease, the underlying inflammation and hypoxia can lead to injury in distant organs (such as the kidneys), subsequently necessitating renal replacement therapy.

(Continued)

Categories	Variables	Group	adjusted variable (yes/no)	DAG paths	Mechanisms by systematic reviews and expert consultations
Clinical features at adimission	NLR	Confounder	Yes	→ Pre-ICU corticosteroid<br>→ IAPA<br>→ Interleukin-6 inhibitors<br>→ Invasive mechanical ventilation<br>→ Renal replacement therapy<br>→ ECMO<br>→ Antifungal prophylaxis<br>→ Broad-spectrum antibiotic	In critically ill patients with influenza, the neutrophil-to-lymphocyte ratio (NLR) at admission, as a key indicator reflecting host inflammatory response and immune dysregulation, is significantly elevated and often suggests a more severe systemic inflammatory state, thereby serving as an important decision-making basis for clinicians to initiate systemic corticosteroid therapy prior to ICU admission [164，165].<br>The elevation of Neutrophil-to-Lymphocyte Ratio (NLR) directly reflects the excessive inflammation and immunosuppression (particularly impaired lymphocyte function) triggered by the influenza virus, which compromises the antifungal immune defenses in the lungs and is one of the mechanisms leading to secondary invasive pulmonary aspergillosis [166].<br>An elevated NLR serves as an indirect marker of a "cytokine storm" (including high levels of IL-6, among others) [167]. When inflammation becomes uncontrolled, the use of IL-6 receptor antagonists (such as tocilizumab) is a direct therapeutic strategy targeting excessive inflammation [168]. Thus, a high NLR can indicate the initiation of such immunomodulatory therapy.<br>A sharp rise in NLR predicts the risk of severe systemic inflammatory response and acute respiratory distress syndrome, which is often the underlying cause of patients developing refractory hypoxemia and ultimately requiring invasive mechanical ventilation support [169,170].<br>Systemic inflammatory response syndrome and sepsis induced by severe influenza can lead to acute kidney injury [151]. As a marker of inflammatory severity, an elevated NLR is associated with the risk of multiple organ dysfunction, including the need for renal replacement therapy [171,172]. When NLR is extremely elevated, it often indicates that the disease has progressed to the most critical stage, with severe respiratory and/or circulatory failure that cannot be corrected by conventional mechanical ventilation, making Extracorporeal Membrane Oxygenation (ECMO) the final life support modality [173].<br>A significantly elevated NLR in severe influenza patients often suggests the possible presence or impending occurrence of bacterial co-infections [174]. Clinically, this typically

					prompts the empirical use of broad-spectrum antibiotics.<br>High NLR reflects the excessive inflammation and immunosuppression triggered by the influenza virus, which compromises the antifungal immune defenses and leads to IAPA [166]. Therefore, a high NLR is an important basis for initiating empirical antifungal prophylaxis [175].<br>**In summary**, in the causal pathway diagram of severe influenza patients, NLR is not an isolated laboratory value but a key hub variable connecting the initial viral infection to adverse clinical outcomes. It can point to the aforementioned factors based on the following coherent logic chain: (1) For the prevention and treatment of complications: The immune deficiency and inflammatory damage predicted by a high NLR directly increase the risk of fatal infections such as secondary pulmonary aspergillosis, thereby driving the need for antifungal prophylaxis or treatment. Simultaneously, it also signals suspected bacterial co-infections, leading to the use of broad-spectrum antibiotics. (2) For the treatment of inflammation itself: NLR serves as a surrogate marker for a cytokine storm. When inflammation is uncontrolled, direct use of immunomodulators like IL-6 inhibitors becomes a therapeutic option. (3) For supportive treatment of organ failure: Extreme inflammatory responses can lead to multi-organ failure, such as in the lungs and kidneys. Consequently, a high NLR is closely associated with the need for invasive mechanical ventilation, renal replacement therapy, and the ultimate life support modality, ECMO. These interventions are both the results of disease severity and may themselves further affect immune status. Therefore, in this DAG, directing arrows from NLR to these factors can clearly delineate a logical pathway from viral infection to immune-inflammatory dysregulation, and then to clinical therapeutic interventions and secondary severe complications.

(Continued)

Categories	Variables	Group	adjusted variable (yes/no)	DAG paths	Mechanisms by systematic reviews and expert consultations
Clinical features at adimission	CRP	Confounder	Yes	→ Pre-ICU corticosteroid<br>→ IAPA<br>→ Interleukin-6 inhibitors<br>→ Invasive mechanical ventilation<br>→ Renal replacement therapy<br>→ ECMO<br>→ Antifungal prophylaxis<br>→ Broad-spectrum antibiotic	In critically ill patients with influenza or severe pneumonia, elevated CRP levels at admission, serving as a key marker of hyperinflammation, have been shown to be a critical determinant in clinicians' decision to initiate corticosteroid therapy [176,177].<br>A significant elevation in CRP indicates a severe systemic inflammatory response [178], which is often associated with immune dysregulation and an increased risk of secondary invasive fungal infections, such as pulmonary aspergillosis [179].<br>IL-6 is the core cytokine that induces hepatic production of CRP [180]. An extremely high CRP level directly reflects the intensity of the IL-6-mediated "cytokine storm." Consequently, it is a significant indicator for clinically considering the use of IL-6 inhibitors, such as tocilizumab, to suppress the excessive inflammatory response [181].<br>CRP levels are closely associated with the severity of viral pneumonia and inflammatory lung injury [182]. Persistently elevated high CRP is a marker of critical illness and potential progression to respiratory failure, thereby influencing the clinician's decision to proceed with invasive mechanical ventilation [183].<br>The systemic hyperinflammatory response induced by severe influenza, manifested as a significant CRP elevation, can lead to multiple organ dysfunction, including acute kidney injury [184]. Thus, high CRP is an indicator for assessing the systemic inflammatory burden and predicting the need for organ support, including renal replacement therapy. Furthermore, an extremely elevated CRP signifies a severe and potentially uncontrolled inflammatory state with organ failure, serving as a reference factor for evaluating whether a patient requires entry into advanced life support protocols, including ECMO [185,186].<br>High CRP indicates a severe systemic inflammatory response [178] and associated with an increased risk of pulmonary aspergillosis [179]. Therefore, a high CRP level can serve as one of the clinical rationales for initiating antifungal prophylaxis.<br>CRP is a commonly used biomarker for judging potential bacterial infections [187]. In severe influenza patients, a markedly elevated CRP level often prompts clinicians to initiate empirical broad-spectrum antibiotic therapy to cover possible bacterial co-infections [188].

					**In summary**, within the causal pathway DAG for severe influenza patients, CRP primarily plays the role of a clinical decision driver or a proxy indicator for disease severity. Specifically, the elevation in CRP directly reflects the intensity of the systemic inflammatory response caused by viral replication and immune system activation, particularly the cytokine storm involving IL-6. This strong inflammatory signal (1) indicates the risk of secondary bacterial or fungal infections, thereby driving the empirical use of broad-spectrum antibiotics or antifungal prophylaxis; (2) itself, as a downstream product of IL-6 pathway activation, becomes a direct rationale for using IL-6 inhibitors for targeted anti-inflammatory therapy; and (3) is closely related to the severity of acute lung injury and multiple organ dysfunction. Therefore, when CRP levels remain significantly and persistently elevated, it predicts that the patient may require more advanced organ support, including invasive mechanical ventilation, renal replacement therapy (due to inflammation-associated kidney injury), and ECMO as an ultimate rescue measure. Hence, when constructing the DAG, it is crucial to understand CRP as a key intermediate variable connecting the pathophysiological state with clinical intervention decisions.

(Continued)

Categories	Variables	Group	adjusted variable (yes/no)	DAG paths	Mechanisms by systematic reviews and expert consultations
Clinical features at adimission	SOFA	Confounder	Yes	→ Pre-ICU corticosteroid<br>→ IAPA<br>→ Interleukin-6 inhibitors<br>→ Invasive mechanical ventilation<br>→ Renal replacement therapy<br>→ ECMO<br>→ Antifungal prophylaxis<br>→ Broad-spectrum antibiotic	SOFA score at ICU admission, as a measure of acute illness severity, influences clinicians' decision to initiate corticosteroid therapy in critically ill patients with severe influenza [189].<br>Upon ICU admission, a high SOFA score suggests the patient has severe immune dysfunction and pulmonary epithelial injury, creating conditions for Aspergillus invasion and thus predisposing to secondary invasive pulmonary aspergillosis [190,191].<br>A high SOFA score indicates a severe inflammatory response and organ injury [76]. In specific contexts (e.g., severe COVID-19), elevated IL-6 levels are associated with poor prognosis, making high-SOFA patients potential candidates for immunomodulatory therapy like interleukin-6 inhibitors [192,193].<br>A high respiratory component score (low PaO_2 /FiO_2 ratio) within the SOFA score is a direct indicator for initiating invasive mechanical ventilation to support failing lung function [194,195].<br>The renal component of the SOFA score is directly based on creatinine and urine output [76]. A high SOFA score implies that acute kidney injury has reached a severity requiring renal replacement therapy [196].<br>When a high SOFA score is primarily driven by refractory respiratory and/or circulatory failure unresponsive to conventional therapy, ECMO serves as the ultimate life-support measure [197,198].<br>High SOFA score suggests the severe immune dysfunction and pulmonary epithelial injury, predisposing to invasive pulmonary aspergillosis [190,191], which may lead to consideration of initiating antifungal prophylaxis.<br>The severe infection/sepsis state represented by a high SOFA score typically triggers a significant rise in CRP, which is an acute-phase reactant protein elevated during infection and inflammation,thereby prompting the initiation of empirical broad-spectrum antibiotic therapy [199].<br>**In summary**, when constructing this DAG, a high SOFA score upon ICU admission points to most factors listed not because it acts as an "etiology" directly causing these events,

					but rather because it serves as a composite indicator of subsequent severe organ dysfunction, playing the following key roles in the causal chain: (1) For measures such as antifungal prophylaxis, broad-spectrum antibiotics, invasive mechanical ventilation, renal replacement therapy, and ECMO, a high SOFA score is a key decision-making criterion per clinical guidelines or protocols for initiating these escalated treatments and life supports. (2) A high SOFA score reflects severe immune dysregulation (e.g., lymphopenia) and organ injury (e.g., disruption of the pulmonary epithelial barrier), which significantly increases the patient's risk of complications such as secondary pulmonary aspergillosis. Simultaneously, it is a strong marker of systemic inflammatory response (elevated CRP, NLR). (3) As a high SOFA score identifies the most critically ill subset among influenza patients, it becomes a crucial indicator for placing them in a single room for enhanced isolation and concentrated care. Therefore, in the causal pathway diagram, arrows from a high SOFA score upon ICU admission to these factors should be interpreted as clinical actions taken based on this severe state, or as high risks and complications accompanying this state.

(Continued)

Categories	Variables	Group	adjusted variable (yes/no)	DAG paths	Mechanisms by systematic reviews and expert consultations
Clinical features at adimission	Bacterial co-infection	Confounder	Yes	→ Pre-ICU corticosteroid<br>→ IAPA<br>→ Invasive mechanical ventilation<br>→ Renal replacement therapy<br>→ ECMO<br>→ Broad-spectrum antibiotic	In patients with community-acquired pneumonia or influenza complicated by bacterial infection, clinicians often initiate corticosteroid therapy due to severe systemic inflammatory response, although this practice remains controversial [200]. A study by Torres et al. demonstrated that adjunctive corticosteroid therapy in patients with severe community-acquired pneumonia reduces treatment failure and mortality, thereby encouraging the consideration of corticosteroids in severely infected patients in clinical practice [201].<br>Bacterial co-infection is significantly associated with IAPA, and the coexistence of both conditions portends a worse prognosis. Feys et al., through an analysis of the pulmonary microbiome in critically ill influenza patients, found that the detection of pulmonary bacterial pathogens was significantly associated with 90-day mortality in IAPA patients, suggesting a potential synergistic pathogenic role between bacterial co-infection and Aspergillus infection [202].<br>Bacterial co-infection is an independent risk factor for the need for invasive mechanical ventilation in patients with severe pneumonia [203]. Rouze et al., in a multicenter European ICU cohort, observed a significantly higher prevalence of early bacterial co-infection among COVID-19 patients receiving invasive ventilation, indicating that bacterial co-infection directly increases the demand for respiratory support [204].<br>Bacterial co-infection can induce sepsis-associated acute kidney injury, thereby increasing the need for renal replacement therapy (RRT)[205]. A study by Mariano et al. showed that sepsis caused by bacterial infection is a major contributor to acute kidney injury requiring RRT, with infection control being closely linked to renal outcomes [206].<br>Bacterial co-infection significantly increases disease severity, leading to an elevated requirement for ECMO support. Kubler et al., in their report on ECMO experience in New Zealand, noted that 54% of patients with viral pneumonia receiving VV-ECMO had bacterial co-infection, suggesting that bacterial co-infection is an important contributing factor to the escalation to ECMO support [207].<br>The diagnosis of bacterial co-infection directly drives the empiric use of broad-spectrum

					antibiotics. In the study by Rouze et al., the definition of bacterial co-infection was based on positive microbiological results, which inevitably lead to the initiation or adjustment of antibiotic therapy, representing the most direct indication for broad-spectrum antibiotic use at ICU admission [208].<br>**In summary**, bacterial co-infection present at ICU admission is a key variable reflecting the patient's pre-admission infectious status, which directly drives multiple downstream clinical events and interventions in the causal pathway. Firstly, bacterial co-infection may prompt clinicians to initiate corticosteroid therapy prior to ICU admission (despite ongoing controversy), thereby linking it to the exposure factor. Secondly, bacterial co-infection itself exhibits a synergistic pathogenic relationship with IAPA, mutually worsening patient prognosis. More importantly, by exacerbating respiratory failure and inducing sepsis, bacterial co-infection significantly increases the need for invasive mechanical ventilation, ECMO, and renal replacement therapy, serving as a critical driver for the escalation of life-support interventions. Concurrently, the diagnosis of bacterial co-infection directly guides the initiation of broad-spectrum antibiotics. Therefore, in the DAG, bacterial co-infection should be directed towards the six factors mentioned above, forming a causal structure that reflects the infection-driven disease progression and treatment decision pathway. This configuration aids in accurately identifying and controlling for confounding, thereby laying the foundation for estimating subsequent causal effects.

(Continued)

Categories	Variables	Group	adjusted variable (yes/no)	DAG paths	Mechanisms by systematic reviews and expert consultations
Clinical features at adimission	Viral co-infection	Confounder	Yes	→ Pre-ICU corticosteroid<br>→ IAPA<br>→ Interleukin-6 inhibitors<br>→ Invasive mechanical ventilation<br>→ Renal replacement therapy<br>→ ECMO<br>→ Antifungal prophylaxis	Viral co-infection may prompt clinicians to use corticosteroids before ICU admission to control excessive inflammatory responses, although this practice remains controversial [209,210]. The severity of viral infection itself may indeed be an indication for initiating corticosteroid therapy.<br>Viral co-infection is an important risk factor for influenza-associated pulmonary aspergillosis. In patients with severe influenza or COVID-19, viral co-infections (such as CMV, EBV, etc.) can further exacerbate immune dysregulation and airway epithelial damage, creating favorable conditions for Aspergillus infection [211]. A case report described a kidney transplant patient with concurrent CMV, influenza virus, and Aspergillus infection, suggesting that multiple viral co-infections may increase the risk of invasive pulmonary aspergillosis [212].<br>Severe cases of viral co-infection are often accompanied by cytokine storms, for which IL-6 inhibitors (e.g., tocilizumab) are used to control excessive inflammation [213]. However, the use of IL-6 inhibitors increases the risk of secondary infections, including viral, bacterial, and fungal infections [214]. This suggests that viral co-infection may serve as a prerequisite for initiating IL-6 inhibitor therapy, while the use of IL-6 inhibitors may in turn increase the risk of other infections.<br>Viral co-infection leads to clinical deterioration and often necessitates invasive mechanical ventilation support. Studies have shown that respiratory viruses are frequently detected in patients with bacterial ventilator-associated pneumonia or hospital-acquired pneumonia, and these patients typically require prolonged mechanical ventilation [215]. In a case of ARDS caused by H7N9 avian influenza co-infected with EBV, the patient's condition progressed rapidly, and conventional mechanical ventilation failed to maintain oxygenation, ultimately requiring VV-ECMO support [216].<br>Viral co-infection can lead to multiple organ failure, including acute kidney injury, thereby necessitating renal replacement therapy [217,218].<br>Severe ARDS caused by viral co-infection is an important indication for initiating

					ECMO support. For instance, the aforementioned patient with H7N9 and EBV co-infection received VV-ECMO due to refractory hypoxemia [219]. This indicates that severe respiratory failure resulting from viral co-infection is a valid indication for ECMO.<br>Given that patients with viral co-infection are at high risk for IAPA, antifungal prophylaxis may be considered in clinical practice. Studies have reported that the incidence of IAPA and CAPA in ICU patients ranges from approximately 10-20%, with high mortality rates [85,220]. However, a randomized controlled trial of posaconazole prophylaxis in critically ill influenza patients showed no significant benefit, suggesting that prophylactic strategies require further optimization [221].<br>**In summary,** Viral co-infection at ICU admission exerts causal effects on multiple downstream factors primarily through pathways involving exacerbated immune dysregulation, promotion of organ dysfunction, and triggering of corresponding therapeutic interventions. Viral co-infection directly increases the risk of IAPA by further compromising airway epithelial integrity and local immune function; concurrently, worsening viral infection often leads to respiratory deterioration requiring invasive mechanical ventilation support, with severe cases necessitating ECMO. Patients with viral co-infection frequently present with bacterial co-infection, leading to increased use of broad-spectrum antibiotics. Additionally, excessive inflammatory responses may prompt the clinical use of IL-6 inhibitors for immunomodulation. Viral co-infection also indirectly influences renal replacement therapy requirements through pathways involving multiple organ dysfunction, and may affect decisions regarding antifungal prophylaxis strategies due to the resulting critical illness state.

(Continued)

Categories	Variables	Group	adjusted variable (yes/no)	DAG paths	Mechanisms by systematic reviews and expert consultations
Interventions at admission	Interleukin-6 inhibitors	Covariate	No	→ IAPA	The use of IL-6 inhibitors may influence the risk of secondary pulmonary aspergillosis through a complex pathway. Inhibiting IL-6 could modulate the immune response [222]. A recent study demonstrates that IAPA is characterized by a state of "severe immune paralysis," with pathway analysis specifically identifying the significant suppression of the IL-6 signaling pathway as a key component of this immunosuppressed state. Crucially, this study shows that therapeutic restoration of immune function—including the reactivation of the IL-6 pathway via an immunostimulant (Pam2ODN)—was associated with dramatically improved survival and fungal clearance in a lethal IAPA model. These findings provide direct experimental evidence that impaired IL-6 signaling is integral to the immune deficit in IAPA. Therefore, it offers a strong mechanistic rationale for the concern that the clinical use of IL-6 inhibitors in severely ill influenza patients could potentially exacerbate this pre-existing "immune paralysis" [223]. Therefore, the clinical application of IL-6 inhibitors for potent anti-inflammatory purposes theoretically carries the potential to further impair the host's antifungal immunity and increase the risk of IAPA [224].
Interventions at admission	Invasive mechanical ventilation	Covariate	No	→ IAPA	Among severe influenza patients receiving invasive mechanical ventilation, clinical management on the first 24 hours of ICU admission prioritizes respiratory support and bacterial infection control, which relatively diminishes attention to antifungal prophylaxis. However, the disruption of the respiratory physical barrier caused by mechanical ventilation, the potential promotion of fungal spore colonization due to positive pressure ventilation, and critical illness-related immunosuppression collectively lead to a significantly increased risk of secondary pulmonary aspergillosis in these patients [225]. Multiple studies have confirmed that invasive mechanical ventilation is an independent risk factor for IAPA [226-228].
Interventions at admission	Renal replacement therapy	Covariate	No	→ IAPA	Patients requiring renal replacement therapy (RRT) often have extremely severe underlying conditions, frequently accompanied by a pronounced systemic inflammatory response syndrome (SIRS) or compensatory anti-inflammatory response syndrome (CARS), leading to severe immunosuppression (i.e., "immune paralysis"), which create a high-risk environment for invasive fungal infections [229,230].

(Continued)

Categories	Variables	Group	adjusted variable (yes/no)	DAG paths	Mechanisms by systematic reviews and expert consultations
Interventions at admission	ECMO	Covariate	No	→ IAPA	In severe influenza patients receiving ECMO, the ECMO circuit itself provides a surface for fungal adherence, while associated critical illness immunosuppression and required anticoagulation significantly increase the risk of invasive fungal infections, making ECMO an independent risk factor for conditions like IAPA [231].
Interventions at admission	Antifungal prophylaxis	Covariate	No	→ IAPA	
Interventions at admission	Broad-spectrum antibiotic	Covariate	No	→ IAPA	The use of broad-spectrum antibiotics is a well-established risk factor for secondary pulmonary aspergillosis (IAPA). The mechanisms involve: (1) Disruption of the microbial barrier: Broad-spectrum antibiotics eliminate bacterial competitors, creating an ecological niche for fungal colonization and invasion; (2) Damage to the mucosal barrier: Certain antibiotics may directly impair the integrity of the respiratory tract mucosa; (3) Immunomodulation: Alterations in the microbiota can affect local immune responses. Therefore, broad-spectrum antibiotics constitute a critical link in the causal chain leading to IAPA [232,233].

(Continued)

Categories	Variables	Group	adjusted variable (yes/no)	DAG paths	Mechanisms by systematic reviews and expert consultations
Others	Admission season	Confounder	No	→ Hemoptysis<br>→ Respiratory rate<br>→ Consolidation<br>→ Bacterial co-infection<br>→ Viral co-infection	Studies have confirmed a significant association between influenza activity and severe hemoptysis (requiring bronchial artery embolization), suggesting an increased risk of hemoptysis events during the influenza season [234].<br>Increased respiratory rate is one of the core indicators of the qSOFA score and has been confirmed as an independent predictor of poor prognosis in influenza patients; such abnormal signs are more common during the influenza season [235].<br>Influenza viral pneumonia often presents as pulmonary infiltrates or consolidation, an imaging finding closely associated with disease severity, and its incidence is significantly higher among hospitalized patients during the influenza season [236].<br>The influenza season is closely related to the incidence of bacterial co-infection. Studies have shown that co-infection with influenza A virus and Staphylococcus aureus increases significantly during peak influenza seasons, and such patients are more likely to require ICU admission [237,238].<br>The seasonal epidemic patterns of respiratory viruses determine the risk of viral co-infection. Studies have shown that the detection rate of co-infection between SARS-CoV-2 and other respiratory viruses is highest during the peak of the influenza season [239-241].<br>**In summary**, admission season, in the DAG, is an upstream exogenous variable. It does not directly determine patients' clinical outcomes but indirectly shapes disease severity and prognostic trajectories by influencing multiple mediating variables. The epidemiological characteristics of the influenza season determine: (1) the risk of viral co-infection, as different respiratory viruses have synchronous or overlapping peaks of circulation; (2) the risk of bacterial co-infection, as influenza virus infection damages the airway epithelium and disrupts immune barriers, leading to a significant increase in secondary bacterial infections during the influenza season; (3) manifestations of local respiratory pathology, including

					pulmonary consolidation and increased respiratory rate; and (4) the risk of severe complications such as hemoptysis. Therefore, admission season should be considered a distal confounder that indirectly affects IAPA outcomes through these clinical and laboratory indicators, rather than a variable that directly points to the outcome.

(Continued)

Categories	Variables	Group	adjusted variable (yes/no)	DAG paths	Mechanisms by systematic reviews and expert consultations
Others	Admission source	Covariate	No	→ Time from influenza symptom onset to ICU	The patient's admission source is not a random or independent characteristic; rather, it profoundly reflects the stage of disease progression, intensity of prior treatment, and baseline severity of illness experienced before ICU admission. These factors collectively determine the speed at which their condition deteriorates to the point of requiring intensive care. Specifically, (1) patients admitted directly from the emergency department typically present with acute, rapid symptom deterioration or are critically ill from the onset, resulting in the shortest interval from symptom onset to the need for ICU intervention. (2) Patients admitted from the community—such as referrals from a family clinic—often follow a relatively gradual disease trajectory, with some experiencing worsening only after outpatient treatment failure, leading to a longer time interval. (3) For patients transferred to the ICU from general wards, the timeline includes an observation and initial treatment period in the ward, as their condition may have progressively deteriorated or complications may have developed before meeting ICU criteria. (4) In the case of patients transferred from other hospitals, the time interval also encompasses the diagnosis and treatment cycle at the initial hospital and the duration of the transfer process, typically making it the longest. Therefore, the admission source serves as a critical triage point, encoding different clinical pathways and backgrounds of illness severity before ICU admission, making it an upstream determinant influencing the key time variable of time from symptom onset to ICU admission. Establishing this directional relationship in this DAG helps more accurately reveal the true causal structure in the process from disease onset to intensive care.
Others	Time from influenza symptom onset to ICU	Covariate	No	-	-

(Continued)

Categories	Variables	Group	adjusted variable (yes/no)	DAG paths	Mechanisms by systematic reviews and expert consultations
Exposure	Pre-ICU corticosteroid	Exposure	NA	→ IAPA<br>→ Antifungal prophylaxis<br>→ Time from influenza symptom onset to ICU	Pre-ICU systemic corticosteroid use is a well-established risk factor for the subsequent development of influenza-associated pulmonary aspergillosis in critically ill patients [242-244]. It is also the simulated causal hypothesis of this study.<br>The initiation of antifungal prophylaxis prior to ICU admission may be influenced by corticosteroid use, as clinicians may empirically prescribe antifungal agents to high-risk patients receiving immunosuppressive therapy, although robust evidence supporting this practice in influenza remains limited [245,246].<br>Corticosteroid administration before ICU admission may delay or alter the clinical trajectory of influenza, potentially prolonging the time from symptom onset to intensive care unit admission due to masking of disease progression or complications [247,248].<br>**In summary**, pre-ICU corticosteroid use serves as the primary exposure and is posited to exert direct effects on the outcome (IAPA), as well as on two intermediate or ancillary variables: antifungal prophylaxis and time from symptom onset to ICU admission. The hypothesized causal pathway from corticosteroids to IAPA is supported by multiple meta-analyses demonstrating a consistent association in critically ill influenza populations [249-251]. The arrow toward antifungal prophylaxis reflects potential clinical practice patterns wherein corticosteroid exposure prompts empirical antifungal initiation, though evidence for the efficacy of this approach in preventing IAPA remains inconclusive [85]. The arrow toward time from symptom onset to ICU accounts for the possibility that corticosteroid administration may alter the natural history of influenza, delaying recognition of severity and subsequent ICU admission, thereby introducing potential time-related confounding [252,253]. Together, these three paths illustrate key downstream influences of pre-ICU corticosteroid use that should be considered in the causal framework to avoid overadjustment bias and ensure valid estimation of the exposure–outcome relationship.
Outcome	IAPA	Outcome	NA	NA	NA

Abbreviations: DAG = directed acyclic graph; BMI = body mass index; NLR = neutrophil-to-lymphocyte ratio; CRP = C-reactive protein; ECMO = extracorporeal membrane oxygenation; HEPA = high-efficiency particulate air; SOFA = sequential organ failure assessment score; IAPA = influenza-associated pulmonary aspergillosis; NA = not available..

References

1. Martin-Loeches I, Lisboa T, Rhodes A, et al. Use of early corticosteroid therapy on ICU admission in patients affected by severe pandemic (H1N1) v influenza A infection[J]. Intensive care medicine, 2011, 37(2): 272-283.
2. Shekhova E, Salazar F, Da Silva Dantas A, Chakraborty T, Wooding EL, White PL, Warris A. Age difference of patients with and without invasive aspergillosis: a systematic review and meta-analysis. BMC Infectious Diseases. 2024 Feb 19;24(1):220.
3. Genitsaridi I, Salpea P, Salim A, et al. of the IDF Diabetes Atlas: global, regional, and national diabetes prevalence estimates for 2024 and projections for 2050[J]. The Lancet Diabetes & Endocrinology, 2026, 14(2): 149-156.
4. Cheemerla S, Balakrishnan M. Global epidemiology of chronic liver disease. Clinical liver disease. 2021 May 1;17(5):365-70.
5. Liu P, Quinn RR, Lam NN, Elliott MJ, Xu Y, James MT, Manns B, Ravani P. Accounting for age in the definition of chronic kidney disease. JAMA internal medicine. 2021 Oct 1;181(10):1359-66.
6. Momtazmanesh S, Moghaddam SS, Ghamari SH, Rad EM, Rezaei N, Shobeiri P, Aali A, Abbasi-Kangevari M, Abbasi-Kangevari Z, Abdelmasseh M, Abdoun M. Global burden of chronic respiratory diseases and risk factors, 1990–2019: an update from the Global Burden of Disease Study 2019. EClinicalMedicine. 2023 May 1;59.
7. Peters A, Nawrot TS, Baccarelli AA. Hallmarks of environmental insults. Cell. 2021 Mar 18;184(6):1455-68.
8. Crooks C J, West J, Morling J R, et al. Respiratory rate responses to both hypercapnia and acidaemia are modified by age in patients with acidosis[J]. Respiratory Physiology & Neurobiology, 2023, 315: 104098.
9. Tobin M J, Chadha T S, Jenouri G, et al. Breathing patterns: 1. Normal subjects[J]. Chest, 1983, 84(2): 202-205.
10. Pellegrino R, Paganelli R, Di Iorio A, et al. Temporal trends, sex differences, and age-related disease influence in neutrophil, lymphocyte count and neutrophil to lymphocyte-ratio: results from InCHIANTI follow-up study[J]. Immunity & Ageing, 2023, 20(1): 46.
11. Lagunas-Rangel F A. Neutrophil-to-lymphocyte ratio in aging: Trends and clinical implications[J]. Experimental Gerontology, 2025: 112908.
12. Ticinesi A, Lauretani F, Nouvenne A, et al. C-reactive protein (CRP) measurement in geriatric patients hospitalized for acute infection[J]. European Journal of Internal Medicine, 2017, 37: 7-12.
13. Sherak R A G, Sajjadi H, Khimani N, et al. SOFA score performs worse than age for predicting mortality in patients with COVID-19[J]. Plos one, 2024, 19(5): e0301013.
14. Liu Y N, Zhang Y F, Xu Q, et al. Infection and co-infection patterns of community-acquired pneumonia in patients of different ages in China from 2009 to 2020: a national surveillance study[J]. The Lancet Microbe, 2023, 4(5): e330-e339.
15. Huang Y, Li S, Ye W, et al. Viral infections in elderly individuals: a comprehensive overview of SARS-CoV-2 and influenza susceptibility, pathogenesis, and clinical treatment strategies[J]. Vaccines, 2025, 13(4): 431.
16. Tylutka A, Walas, Zembron-Lacny A. Level of IL-6, TNF, and IL-1β and age-related diseases: a systematic review and meta-analysis[J]. Frontiers in immunology, 2024, 15: 1330386.
17. Michels-Zetsche J D, Röser E, Ersöz H, et al. Influence of age on outcomes in prolonged weaning from mechanical ventilation[J]. BMJ Open Respiratory Research, 2025, 12(1).
18. Kiekkas P, Tzenalis A, Gklava V, Stefanopoulos N, Voyagis G, Aretha D. Delayed Admission to the Intensive Care Unit and Mortality of Critically Ill Adults: Systematic Review and Meta-Analysis. BioMed research international. 2022;2022(1):4083494.
19. Ahn YH, Lee J, Oh DK, Lee SY, Park MH, Lee H, Lim CM, Lee SM, Lee HY. Association between the timing of ICU admission and mortality in patients with hospital-onset sepsis: a

nationwide prospective cohort study. Journal of intensive care. 2023 Apr 21;11(1):16."
20. Segaloff H E, Evans R, Arshad S, et al. The impact of obesity and timely antiviral administration on severe influenza outcomes among hospitalized adults[J]. Journal of medical virology, 2018, 90(2): 212-218.
21. Ohba H, Miwa S, Shirai M, Kanai M, Eifuku T, Suda T, Hayakawa H, Chida K. Clinical characteristics and prognosis of chronic pulmonary aspergillosis. Respiratory medicine. 2012 May 1;106(5):724-9.
22. Hanson C, Rutten EP, Wouters EF, Rennard S. Influence of diet and obesity on COPD development and outcomes. International journal of chronic obstructive pulmonary disease. 2014 Aug 5:723-33.
23. Guo N, Shi H, Zhao H, et al. Causal relationships of lifestyle behaviours and body fat distribution on diabetic microvascular complications: a Mendelian randomization study[J]. Frontiers in Genetics, 2024, 15: 1381322.
24. Quek J, Chan KE, Wong ZY, Tan C, Tan B, Lim WH, Tan DJ, Tang AS, Tay P, Xiao J, Yong JN. Global prevalence of non-alcoholic fatty liver disease and non-alcoholic steatohepatitis in the overweight and obese population: a systematic review and meta-analysis. The lancet Gastroenterology & hepatology. 2023 Jan 1;8(1):20-30.
25. Tan DJ, Ng CH, Lin SY, Pan XH, Tay P, Lim WH, Teng M, Syn N, Lim G, Yong JN, Quek J. Clinical characteristics, surveillance, treatment allocation, and outcomes of non-alcoholic fatty liver disease-related hepatocellular carcinoma: a systematic review and meta-analysis. The Lancet Oncology. 2022 Apr 1;23(4):521-30.
26. McGlynn KA, Petrick JL, El-Serag HB. Epidemiology of hepatocellular carcinoma. Hepatology. 2021 Jan;73:4-13.
27. Stasi A, Cosola C, Caggiano G, Cimmarusti MT, Palieri R, Acquaviva PM, Rana G, Gesualdo L. Obesity-related chronic kidney disease: principal mechanisms and new approaches in nutritional management. Frontiers in nutrition. 2022 Jun 24;9:925619.
28. Hall JE, do Carmo JM, da Silva AA, Wang Z, Hall ME. Obesity, kidney dysfunction and hypertension: mechanistic links. Nature reviews nephrology. 2019 Jun;15(6):367-85.
29. Gibson G. Obesity, respiratory function and breathlessness. Thorax. 2000 Aug;55(Suppl 1):S41.
30. Peters U, Suratt BT, Bates JH, Dixon AE. Beyond BMI: obesity and lung disease. Chest. 2018 Mar 1;153(3):702-9.
31. Mannino DM, Reichert MM, Davis KJ. Lung function decline and outcomes in an adult population. American journal of respiratory and critical care medicine. 2006 May 1;173(9):985-90.
32. Kwong J C, Campitelli M A, Rosella L C. Obesity and respiratory hospitalizations during influenza seasons in Ontario, Canada: a cohort study[J]. Clinical Infectious Diseases, 2011, 53(5): 413-421.
33. Deng Q Z, Hu A R, Li H S. Clinical analysis of 42 obese patients with 2009 pandemic influenza A (H1N1)[J]. Dis Surveil, 2010, 25(9): 713-715.
34. Falagas M E, Kompoti M. Obesity and infection[J]. The Lancet infectious diseases, 2006, 6(7): 438-446.
35. Zhao S S. IL-6 receptor inhibition is not associated with body weight or composition: Mendelian randomisation study[J]. Clinical Rheumatology, 2024, 43(4): 1411-1412.
36. Bagshaw S M, Sood M M, Long J, et al. Acute kidney injury among critically ill patients with pandemic H1N1 influenza A in Canada: cohort study[J]. BMC nephrology, 2013, 14(1): 123
37. Abdallah S, Abudayah A. 49: Challenges during VV-ECMO in a severely obese patient with Influenza-A[J]. ASAIO Journal, 2025, 71(Supplement 6): 9.
38. Rawal G, Kumar R, Yadav S, et al. H1N1 influenza induced acute respiratory distress syndrome rescued by extracorporeal membrane oxygenation: A case report[J]. Journal of Translational Internal Medicine, 2017, 5(3): 182-185.
39. Pourbaix A, Lafont Rapnouil B, Guéry R, Lanternier F, Lortholary O, Cohen JF. Smoking as a risk factor of invasive fungal disease: systematic review and meta-analysis. Clinical Infectious Diseases. 2020 Aug 14;71(4):1106-19.

40. Ryu JH, Colby TV, Hartman TE, Vassallo R. Smoking-related interstitial lung diseases: a concise review. European Respiratory Journal. 2001 Jan 1;17(1):122-32.
41. Chalmers GW, Macleod KJ, Little SA, Thomson LJ, McSharry CP, Thomson NC. Influence of cigarette smoking on inhaled corticosteroid treatment in mild asthma. Thorax. 2002 Mar 1;57(3):226-30.
42. Sonnex K, Alleemudder H, Knaggs R. Impact of smoking status on the efficacy of inhaled corticosteroids in chronic obstructive pulmonary disease: a systematic review. BMJ open. 2020 Apr 1;10(4):e037509.
43. American Diabetes Association. 5. Facilitating behavior change and well-being to improve health outcomes: standards of medical care in diabetes—2021[J]. Diabetes care, 2021, 44(Supplement_1): S53-S72.
44. Caturegli G, Zhu X, Palis B, Mullett TW, Resio BJ, Boffa DJ. Smoking Status of the US Cancer Population and a New Perspective From the National Cancer Database. JAMA oncology. 2025 May 1;11(5):564-6.
45. Yuan S, Chen J, Ruan X, Sun Y, Zhang K, Wang X, Li X, Gill D, Burgess S, Giovannucci E, Larsson SC. Smoking, alcohol consumption, and 24 gastrointestinal diseases: Mendelian randomization analysis. elife. 2023 Feb 2;12:e84051.
46. El-Zayadi AR. Heavy smoking and liver. World journal of gastroenterology: WJG. 2006 Oct 14;12(38):6098.
47. Orth SR, Hallan SI. Smoking: a risk factor for progression of chronic kidney disease and for cardiovascular morbidity and mortality in renal patients—absence of evidence or evidence of absence?. Clinical Journal of the American Society of Nephrology. 2008 Jan 1;3(1):226-36.
48. Doccioli C, Sera F, Francavilla A, Cupisti A, Biggeri A. Association of cadmium environmental exposure with chronic kidney disease: A systematic review and meta-analysis. Science of The Total Environment. 2024 Jan 1;906:167165.
49. Labaki WW, Han MK. Chronic respiratory diseases: a global view. The Lancet Respiratory Medicine. 2020 Jun 1;8(6):531-3.
50. Kaminski T W, Brzoska T, Li X, et al. Lung microvascular occlusion by platelet-rich neutrophil-platelet aggregates promotes cigarette smoke–induced severe flu[J]. JCI insight, 2024, 9(2): e167299.
51. Wu X, Li R F, Lin Z S, et al. Coinfection with influenza virus and non-typeable Haemophilus influenzae aggregates inflammatory lung injury and alters gut microbiota in COPD mice[J]. Frontiers in Microbiology, 2023, 14: 1137369.
52. Bucher H, Duechs M J, Tilp C, et al. Tiotropium Attenuates Virus-Induced Pulmonary Inflammation in Cigarette Smoke–Exposed Mice[J]. The Journal of Pharmacology and Experimental Therapeutics, 2016, 357(3): 606-618.
53. Alroumi F, Abdul Azim A, Kergo R, et al. The impact of smoking on patient outcomes in severe sepsis and septic shock[J]. Journal of intensive care, 2018, 6(1): 42.
54. Van Zyl-Smit R N, Brunet L, Pai M, et al. The convergence of the global smoking, COPD, tuberculosis, HIV, and respiratory infection epidemics[J]. Infectious disease clinics of North America, 2010, 24(3): 693.
55. Wu X, Li R F, Lin Z S, et al. Coinfection with influenza virus and non-typeable Haemophilus influenzae aggregates inflammatory lung injury and alters gut microbiota in COPD mice[J]. Frontiers in Microbiology, 2023, 14: 1137369.
56. Sparks J A, Brennan D C, Lawrence S J. Smoking association with influenza infection in renal transplant recipients[J]. Transplant Infectious Disease, 2014, 16(1): 153-157.
57. Rosoff D B, Yoo J, Lohoff F W. Smoking is significantly associated with increased risk of COVID-19 and other respiratory infections[J]. Communications biology, 2021, 4(1): 1230.
58. Parisi C E, Yousey-Hindes K, Holstein R, et al. Drug use and severe outcomes among adults hospitalized with influenza, 2016–2019[J]. Influenza and Other Respiratory Viruses, 2023, 17(1): e13052.

59. Kaminski T W, Brzoska T, Li X, et al. Lung microvascular occlusion by platelet-rich neutrophil-platelet aggregates promotes cigarette smoke–induced severe flu[J]. JCI insight, 2024, 9(2): e167299.
60. Ahmadikia K, Hashemi S J, Khodavaisy S, et al. The double-edged sword of systemic corticosteroid therapy in viral pneumonia: A case report and comparative review of influenza-associated mucormycosis versus COVID-19 associated mucormycosis[J]. Mycoses, 2021, 64(8): 798-808.
61. Fernández-Trujillo L, Eraso I, Morales E I, et al. Invasive aspergillosis in a patient with diabetes mellitus as the only risk factor: case report and literature review[J]. Journal of investigative medicine high impact case reports, 2023, 11: 23247096231175443.
62. Ahmed S, Hameed M, Al-Tikriti M, et al. Etiology, diagnostic modalities, and short-term outcomes of hemoptysis—a retrospective cross-sectional study from a tertiary care center in Qatar[J]. Journal of Thoracic Disease, 2023, 15(12): 6634.
63. Chen H, Wu C, Cao L, et al. The association between the neutrophil-to-lymphocyte ratio and type 2 diabetes mellitus: a cross-sectional study[J]. BMC Endocrine Disorders, 2024, 24(1): 107.
64. Stanimirovic J, Radovanovic J, Banjac K, et al. Role of C-reactive protein in diabetic inflammation[J]. Mediators of inflammation, 2022, 2022(1): 3706508.
65. Hua Y, Ding N, Jing H, et al. Association between SOFA score and risk of acute kidney injury in patients with diabetic ketoacidosis: an analysis of the MIMIC-IV database[J]. Frontiers in Endocrinology, 2024, 15: 1462330.
66. Darwitz B P, Genito C J, Thurlow L R. Triple threat: how diabetes results in worsened bacterial infections[J]. Infection and immunity, 2024, 92(9): e00509-23.
67. Mazumder M K, Mozazfia K T E, Ahmed M, et al. Admission glycemic gap and other glycemic indices in assessing the need for mechanical ventilation among neurocritical patients with diabetes mellitus[J]. Bangabandhu Sheikh Mujib Medical University Journal, 2025, 18(2): e77770.
68. Vasbinder A, Anderson E, Shadid H, et al. Inflammation, hyperglycemia, and adverse outcomes in individuals with diabetes mellitus hospitalized for COVID-19[J]. Diabetes care, 2022, 45(3): 692-700.
69. Br VK, Sarin SK. Acute-on-chronic liver failure: terminology, mechanisms and management. Clinical and Molecular Hepatology. 2023 Mar 20;29(3):670.
70. Luo J, Li J, Li P, Liang X, Hassan HM, Moreau R, Li J. Acute-on-chronic liver failure: Far to go—A review. Critical Care. 2023 Jul 1;27(1):259.
71. Chen J, Yang Q, Huang J, Li L. Risk factors for invasive pulmonary aspergillosis and hospital mortality in acute-on-chronic liver failure patients: a retrospective-cohort study. International Journal of Medical Sciences. 2013 Sep 18;10(12):1625.
72. Lahmer T, Peçanha-Pietrobom PM, Schmid RM, Colombo AL. Invasive fungal infections in acute and chronic liver impairment: a systematic review. Mycoses. 2022 Feb;65(2):140-51.
73. Soulaidopoulos S, Goulis I, Cholongitas E. Pulmonary manifestations of chronic liver disease: a comprehensive review. Annals of Gastroenterology. 2020 Mar 27;33(3):237.
74. Kwo PY. Shortness of breath in the patient with chronic liver disease. Clinics in Liver Disease. 2012 May 1;16(2):321-9.
75. Sureka B, Bansal K, Patidar Y, Kumar S, Arora A. Thoracic perspective revisited in chronic liver disease. Gastroenterology Report. 2015 Aug 1;3(3):194-200.
76. Lambden S, Laterre PF, Levy MM, Francois B. The SOFA score—development, utility and challenges of accurate assessment in clinical trials. Critical Care. 2019 Nov 27;23(1):374.
77. Lee D U, Fan G H, Hastie D J, et al. The clinical impact of cirrhosis on the hospital outcomes of patients admitted with influenza infection: propensity score matched analysis of 2011–2017 US hospital data[J]. Journal of Clinical and Experimental Hepatology, 2021, 11(5): 531-543.
78. Karvellas CJ, Gustot T, Fernandez J. Management of the acute on chronic liver failure in the intensive care unit. Liver International. 2025 Mar;45(3):e15659.
79. Karvellas CJ, Bajaj JS, Kamath PS, Napolitano L, O'Leary JG, Solà E, Subramanian R, Wong F, Asrani SK. AASLD Practice Guidance on Acute-on-chronic liver failure and the management of critically ill patients with cirrhosis. Hepatology. 2024 Jun 1;79(6):1463-502.

80. Dobrilovic N, March R, Yin K, Lateef O, Alimohamed M, Bak E, Delibasic M, Karlson K, Edwards N, Raman J. Liver dysfunction associated with in-hospital mortality in adult extracorporeal membrane oxygenation support. Critical Care Explorations. 2021 Jul 1;3(7):e0484.
81 .Premkumar M, Devurgowda D, Dudha S, Maiwall R, Bihari C, Grover S, Gupta E, Kumar S, Sarin SK. A/H1N1/09 influenza is associated with high mortality in liver cirrhosis. Journal of clinical and experimental hepatology. 2019 Mar 1;9(2):162-70.
82. Bianchi FP, Losito F, Labarile N, Shahini E, Cozzolongo R. Prevention of influenza complications in patients with liver disease: a retrospective cohort study. Frontiers in Public Health. 2023 Dec 20;11:1288126.
83. Duchini A, Viernes M E, Nyberg L M, et al. Hepatic decompensation in patients with cirrhosis during infection with influenza A[J]. Archives of internal medicine, 2000, 160(1): 113-115
84. Barros N, Rosenblatt RE, Phipps MM, Fomin V, Mansour MK. Invasive fungal infections in liver diseases. Hepatology Communications. 2023 Sep 1;7(9):e0216.
85. Feys S, Carvalho A, Clancy CJ, Gangneux JP, Hoenigl M, Lagrou K, Rijnders BJ, Seldeslachts L, Vanderbeke L, van de Veerdonk FL, Verweij PE. Influenza-associated and COVID-19-associated pulmonary aspergillosis in critically ill patients. The Lancet Respiratory Medicine. 2024 Sep 1;12(9):728-42
86. Patel VC, Williams R. Antimicrobial resistance in chronic liver disease. Hepatology international. 2020 Jan;14(1):24-34.
87. Rivedal M, Haaskjold YL, Eikrem Ø, Bjørneklett R, Marti HP, Knoop T. Use of corticosteroids in Norwegian patients with immunoglobulin a nephropathy progressing to end-stage kidney disease: a retrospective cohort study. BMC nephrology. 2024 Jan 29;25(1):42.
88. Caiazzo E, Rezig AO, Bruzzese D, Ialenti A, Cicala C, Cleland JG, Guzik TJ, Maffia P, Pellicori P. Systemic administration of glucocorticoids, cardiovascular complications and mortality in patients hospitalised with COVID-19, SARS, MERS or influenza: A systematic review and meta-analysis of randomised trials. Pharmacological Research. 2022 Feb 1;176:106053.
89. Kallay N, Dunagan DP, Adair N, Chin R, Haponik EF. Hemoptysis in patients with renal insufficiency: the role of flexible bronchoscopy. Chest. 2001 Mar 1;119(3):788-94.
90. Xu Y, Chen Y, Mai X, et al. Prognostic value of neutrophil-to-lymphocyte ratio for the clinical outcomes of chronic kidney diseases: an update systematic review and meta-analysis[J]. BMC nephrology, 2025, 26(1): 419.
91. Stenvinkel P, Lindholm B. C-reactive protein in end-stage renal disease: are there reasons to measure it?[J]. Blood purification, 2004, 23(1): 72-78.
92. Li J, Chen J, Lan H, et al. Role of C-reactive protein in kidney diseases[J]. Kidney Diseases, 2023, 9(2): 73-81.
93. Ceprián N, Martínez de Toda I, Caro P J, et al. An immuno-fragile profile is associated with mortality risk in patients with chronic kidney disease[J]. Biomedicines, 2025, 13(10): 2370.
94. Naqvi S B, Collins A J. Infectious complications in chronic kidney disease[J]. Advances in chronic kidney disease, 2006, 13(3): 199-204.
95. Sagedal S, Hovd M, Åsberg A, et al. Increased levels of ficolin-1 and of C3dg are independently associated with high risk of infection in patients with chronic kidney disease: a prospective cohort study[J]. Frontiers in Immunology, 2025, 16: 1645347.
96. Pecoits-Filho R, Lindholm B, Axelsson J, Stenvinkel P. Update on interleukin-6 and its role in chronic renal failure. Nephrology Dialysis Transplantation. 2003 Jun 1;18(6):1042-5.
97. Frolova NF, Tomilina NA, Usatyuk SS, Dyakova EN, Sysoeva IL, Terentyeva NV, Frolov MA, Kim IG. Interleukin-6 receptor inhibitor levilimab in the treatment of COVID-19 in patients with chronic kidney disease. Clinical nephrology. 2023 May 20;15(1):21-9.
98. Vemuri SV, Rolfsen ML, Sykes AV, Takiar PG, Leonard AJ, Malhotra A, Spragg RG, Macedo E, Hepokoski ML. Association between acute kidney injury during invasive mechanical ventilation and ICU outcomes and respiratory system mechanics. Critical care explorations. 2022 Jul 1;4(7):e0720.
99. Ng PY, Ip A, Ng AK, Sin SW, Chan JF, To KK, Yap DY. Risk of acute kidney injury in critically-ill patients with COVID-19 compared with seasonal influenza: a retrospective cohort study. EClinicalMedicine. 2024 Apr 1;70.

100. Cau A, Cheng MP, Lee T, Levin A, Lee TC, Vinh DC, Lamontagne F, Singer J, Walley KR, Murthy S, Patrick D. Acute kidney injury and renal replacement therapy in COVID-19 versus other respiratory viruses: a systematic review and meta-analysis. Canadian Journal of Kidney Health and Disease. 2021 Oct;8:20543581211052185.
101. Villa G, Katz N, Ronco C. Extracorporeal membrane oxygenation and the kidney. Cardiorenal medicine. 2015 Oct 17;6(1):50-60.
102. Guru PK, Balasubramanian P, Ghimire M, Bohman JK, Seelhammer TG, Kashani KB, Schears GJ. Acute kidney injury in patients before and after extracorporeal membrane oxygenation (ECMO)–Retrospective longitudinal analysis of the hospital outcomes. Journal of Critical Care. 2024 Jun 1;81:154528.
103. Su G, Iwagami M, Qin X, McDonald H, Liu X, Carrero JJ, Stålsby Lundborg C, Nitsch D. Kidney disease and mortality in patients with respiratory tract infections: a systematic review and meta-analysis. Clinical kidney journal. 2021 Feb 1;14(2):602-11.
104. Lamoth F, Albrich WC, Ragozzino S, Bosetti D, Delaloye J, El Khoury C, Munting A, Portillo V, Reinhold I, Sumer J, Zbinden A. Management of Invasive Pulmonary Aspergillosis in Intensive Care Units: Guidelines From the Fungal Infection Network of Switzerland (FUNGINOS). Mycoses. 2025 Nov;68(11):e70132.
105. Lamoth F, Calandra T. Pulmonary aspergillosis: diagnosis and treatment. European Respiratory Review. 2022 Nov 29;31(166).
106. Su G, Xu H, Riggi E, He Z, Lu L, Lindholm B, Marrone G, Wen Z, Liu X, Johnson DW, Carrero JJ. Association of kidney function with infections by multidrug-resistant organisms: an electronic medical record analysis. Scientific reports. 2018 Sep 6;8(1):13372.
107. Eyler RF, Mueller BA. Antibiotic dosing in critically ill patients with acute kidney injury. Nature Reviews Nephrology. 2011 Apr;7(4):226-35.
108. Chellappan DK, Prasher P, Shukla SD, Yee TW, Kah TK, Xyan TW, Kid TW, Si TH, Weng TS, Molugulu N, Sakthivel LP. Exploring the role of antibiotics and steroids in managing respiratory diseases. Journal of Biochemical and Molecular Toxicology. 2022 Oct;36(10):e23174.
109. Leung JM, Tiew PY, Mac Aogáin M, Budden KF, Yong VF, Thomas SS, Pethe K, Hansbro PM, Chotirmall SH. The role of acute and chronic respiratory colonization and infections in the pathogenesis of COPD. Respirology. 2017 May;22(4):634-50.
110. O'Gurek D, Choi HY. Hemoptysis: evaluation and management. American family physician. 2022 Feb;105(2):144-51.
111. Beltramo G, Cottenet J, Mariet AS, Georges M, Piroth L, Tubert-Bitter P, Bonniaud P, Quantin C. Chronic respiratory diseases are predictors of severe outcome in COVID-19 hospitalised patients: a nationwide study. European Respiratory Journal. 2021 Dec 9;58(6).
112. Wang N, Chi Y, Wang Q, Long Y, Liu D, Zhao Z, He H. Relationship between lung consolidation size measured by ultrasound and outcome in ICU patients with respiratory failure. BMC Pulmonary Medicine. 2025 Dec;25(1):1-7.
113. HSU A C Y, See H V, Hansbro P M, et al. Innate immunity to influenza in chronic airways diseases[J]. Respirology, 2012, 17(8): 1166-1175.
114. Paliogiannis P, Fois A G, Sotgia S, et al. The neutrophil-to-lymphocyte ratio as a marker of chronic obstructive pulmonary disease and its exacerbations: A systematic review and meta-analysis[J]. European Journal of Clinical Investigation, 2018, 48(8): e12984.
115. Wang T, Duan W, Jia X, et al. Associations of combined phenotypic ageing and genetic risk with incidence of chronic respiratory diseases in the UK Biobank: a prospective cohort study[J]. European Respiratory Journal, 2024, 63(2).
116. Ji S, Dai MY, Huang Y, Ren XC, Jiang ML, Qiao JP, Zhang WY, Xu YH, Shen JL, Zhang RQ, Fei GH. Influenza a virus triggers acute exacerbation of chronic obstructive pulmonary disease by increasing proinflammatory cytokines secretion via NLRP3 inflammasome activation. Journal of Inflammation. 2022 Jun 23;19(1):8.
117. Wu X, Li R F, Lin Z S, et al. Coinfection with influenza virus and non-typeable Haemophilus influenzae aggregates inflammatory lung injury and alters gut microbiota in COPD mice[J]. Frontiers in Microbiology, 2023, 14: 1137369.
118. Smith C B, Golden C, Klauber M R, et al. Interactions between viruses and bacteria in patients with chronic bronchitis[J]. Journal of Infectious Diseases, 1976, 134(6): 552-561

119. Moghoofei M, Mostafaei S, Kondori N, et al. Bacterial and viral coinfection in idiopathic pulmonary fibrosis patients: the prevalence and possible role in disease progression[J]. BMC pulmonary medicine, 2022, 22(1): 60.
120. Luque-Paz D, Tattevin P, Loubet P, et al. Chronic use of inhaled corticosteroids in patients admitted for respiratory virus infections: a 6-year prospective multicenter study[J]. Scientific Reports, 2022, 12(1): 4199.
121. Stefanska I, Romanowska M, Donevski S, et al. Co-infections with influenza and other respiratory viruses[M]//Respiratory Regulation-The Molecular Approach. Dordrecht: Springer Netherlands, 2012: 291-301.
122. Marwah V, Jyothis MC, Choudhary R, Shrinath V. Successful management of cytokine storm induced acute respiratory distress syndrome secondary to H1N1 influenza with tocilizumab. Medical Journal of Dr. DY Patil University. 2023 Jul 1;16(4):648-50.
123. Stickland MK, Neder JA, Guenette JA, O'Donnell DE, Jensen D. Using cardiopulmonary exercise testing to understand dyspnea and exercise intolerance in respiratory disease. Chest. 2022 Jun 1;161(6):1505-16.
124. Mulpuru S, Andrew MK, Ye L, Hatchette T, LeBlanc J, El-Sherif M, MacKinnon-Cameron D, Aaron SD, Alvarez GG, Forster AJ, Ambrose A. Impact of respiratory viral infections on mortality and critical illness among hospitalized patients with chronic obstructive pulmonary disease. Influenza and Other Respiratory Viruses. 2022 Nov;16(6):1172-82.
125. Scholz H, Boivin FJ, Schmidt-Ott KM, Bachmann S, Eckardt KU, Scholl UI, Persson PB. Kidney physiology and susceptibility to acute kidney injury: implications for renoprotection. Nature Reviews Nephrology. 2021 May;17(5):335-49.
126. Pelaia C, Pastori D, Armentaro G, Miceli S, Cassano V, Barbara K, Pelaia G, Perticone M, Maio R, Pignatelli P, Violi F. Predictors of renal function worsening in patients with chronic obstructive pulmonary disease (COPD): a multicenter observational study. Nutrients. 2021 Aug 16;13(8):2811.
127. Zakrajsek JK, Min SJ, Ho PM, Kiser TH, Kannappan A, Sottile PD, Allen RR, Althoff MD, Reynolds PM, Moss M, Burnham EL. Extracorporeal membrane oxygenation for refractory asthma exacerbations with respiratory failure. Chest. 2023 Jan 1;163(1):38-51.
128. Ji S, Dai MY, Huang Y, Ren XC, Jiang ML, Qiao JP, Zhang WY, Xu YH, Shen JL, Zhang RQ, Fei GH. Influenza a virus triggers acute exacerbation of chronic obstructive pulmonary disease by increasing proinflammatory cytokines secretion via NLRP3 inflammasome activation. Journal of Inflammation. 2022 Jun 23;19(1):8.
129. Bassetti M, Leon C, Timsit JF. Are prophylactic antifungals in highly colonized patients safe and effective?. Intensive Care Medicine. 2015 Jul;41(7):1336-9.
130. Hunt A E, Sop J, Kahre J. Diffuse Alveolar Hemorrhage: A Rare Life-Threatening Cause of Massive Hemoptysis[J]. Cureus, 2024, 16(7).
131. Zheng J, Huo X, Huai Y, et al. Epidemiology, seasonality and treatment of hospitalized adults and adolescents with influenza in Jingzhou, China, 2010-2012[J]. PLoS One, 2016, 11(3): e0150713.
132. Wu TH, Lee CY, Huang YC. Case 18. A 9-Year-Old Girl with Fever, Cough Followed by Hemoptysis and Dyspnea: Severe Influenza Virus Infections. InPaediatric Infectious Diseases: A practical guide and cases 2023 Feb 16 (pp. 85-91). Singapore: Springer Nature Singapore.
133. Pasula S, Chandrasekar P. Spontaneous hemoptysis in a patient with COVID-19. Chest. 2021 Jul 1;160(1):e39-44.
134. Liu S, Li Z, Zheng J, He N. Invasive Aspergillus outbreak in inhalation injury: a case presentation and literature review. BMC Infectious Diseases. 2022 Apr 18;22(1):386.
135. Obar JJ, Shepardson KM. Coinfections in the lung: How viral infection creates a favorable environment for bacterial and fungal infections. Plos Pathogens. 2023 May 4;19(5):e1011334.
136. Salazar F, Bignell E, Brown GD, Cook PC, Warris A. Pathogenesis of respiratory viral and fungal coinfections. Clinical Microbiology Reviews. 2022 Jan 19;35(1):e00094-21.
137. Deshwal H, Sinha A, Mehta AC. Life-threatening hemoptysis. Seminars in Respiratory and Critical Care Medicine. 2021; 42(01): 145-159.
138. Takizawa D, Ishida T, Nakano H, Tachi H, Yamamoto Y, Shimizu K, Iizumi T, Sumiya T, Ohnishi K, Sakurai H. A case of massive hemoptysis caused by lung cancer saved by VV

ECMO and hemostasis achieved by radiotherapy. International Cancer Conference Journal 2024 Jan (Vol. 13, No. 1, pp. 54-57).

139. Li H, Yang S, Gu L, et al. Effect of low-to-moderate-dose corticosteroids on mortality of hospitalized adolescents and adults with influenza A (H1N1) pdm09 viral pneumonia[J]. Influenza and other respiratory viruses, 2017, 11(4): 345-354.
140. Cao B, Gao H, Zhou B, et al. Adjuvant corticosteroid treatment in adults with influenza A (H7N9) viral pneumonia[J]. Critical care medicine, 2016, 44(6): e318-e328.
141. Villgran VD, Lyons C, Nasrullah A, Abalos CC, Bihler E, Alhajhusain A. Acute respiratory failure. Critical care nursing quarterly. 2022 Jul 1;45(3):233-47.
142. Tian J, Liu C, Wang X, Zhang L, Zhong G, Huang G, Wang H, Hu H, Gong L, Liu D. Comparative analysis of clinical features of lower respiratory tract infection with respiratory syncytial virus and influenza virus in adults: a retrospective study. BMC Pulmonary Medicine. 2023 Sep 15;23(1):350.
143. Lee WC, Chang CC, Ho MC, Lin CM, Leu SW, Lin CK, Fang YH, Huang SY, Lin YC, Chuang MC, Yang TM. Invasive pulmonary aspergillosis among patients with severe community-acquired pneumonia and influenza in ICUs: a retrospective cohort study. Pneumonia. 2024 May 25;16(1):10.
144. Raghav H, Tayal P, Das R, Mehta DK. Appropriate use of antibiotics for the management of respiratory tract infections. Infectious Disorders-Drug TargetsDisorders). 2022 Aug 1;22(5):36-46.
145. Machado-Curbelo C, González-Quevedo A. Hypoxemia and cytokine storm in COVID-19: clinical implications. MEDICC review. 2022 Nov 4;23:54-9.
146. Kang S, Kishimoto T. Interplay between interleukin-6 signaling and the vascular endothelium in cytokine storms. Experimental & molecular medicine. 2021 Jul;53(7):1116-23.
147. Dupuis C, Bouadma L, de Montmollin E, Goldgran-Toledano D, Schwebel C, Reignier J, Neuville M, Ursino M, Siami S, Ruckly S, Alberti C. Association between early invasive mechanical ventilation and day-60 mortality in acute hypoxemic respiratory failure related to coronavirus disease-2019 pneumonia. Critical Care Explorations. 2021 Jan 1;3(1):e0329.
148. Strohbehn IA, Zhao S, Seethapathy H, Lee M, Rusibamayila N, Allegretti AS, Parada XV, Sise ME. Acute kidney injury incidence, recovery, and long-term kidney outcomes among hospitalized patients with COVID-19 and influenza. Kidney International Reports. 2021 Oct 1;6(10):2565-74.
149. Robert A, Honoré PM, Bulpa P, Michaux I. Managing refractory hypoxemia in acute respiratory distress syndrome obese patients with veno-venous extra-corporeal membrane oxygenation: a narrative review. Journal of clinical medicine. 2025 Feb 28;14(5):1653.
150. Patel YJ, Gannon WD, Francois SA, Stokes JW, Tipograf Y, Landsperger JS, Semler MW, Casey JD, Rice TW, Bacchetta M. Extracorporeal membrane oxygenation circuits in parallel for refractory hypoxemia in patients with COVID-19. The Journal of Thoracic and Cardiovascular Surgery. 2024 Feb 1;167(2):746-54.
151. Li W, Gao M, Hao Y, et al. Beneficial effect of sequential treatment with high-dose steroids and short-course oral glucocorticoids in patients with severe influenza virus-associated pneumonia[J]. Scientific Reports, 2024, 14(1): 25023.
152. Vanderbeke L, Janssen NAF, Bergmans DCJJ, et al. Posaconazole for prevention of invasive pulmonary aspergillosis in critically ill influenza patients (POSA-FLU): a randomised, open-label, proof-ofconcept trial. Intensive Care Med. 2021;47(6):674-686.
153. Tobin JM, Nickolich KL, Ramanan K, Pilewski MJ, Lamens KD, Alcorn JF, Robinson KM. Influenza suppresses neutrophil recruitment to the lung and exacerbates secondary invasive pulmonary aspergillosis. The Journal of Immunology. 2020 Jul 15;205(2):480-8.
154. Lu LY, Lee HM, Burke A, Bassi GL, Torres A, Fraser JF, Fanning JP. Prevalence, risk factors, clinical features, and outcome of influenza-associated pulmonary aspergillosis in critically ill patients: a systematic review and meta-analysis. Chest. 2024 Mar 1;165(3):540-58.
155. Mathis G, Beckh S, Görg C, Mathis G, Beckh S, Mathis G, Görg C, Mathis G. Lung consolidation. Chest sonography. 2011 Jul 21; 55-114.
156. Gu Y, Zuo X, Zhang S, Ouyang Z, Jiang S, Wang F, Wang G. The mechanism behind influenza virus cytokine storm. Viruses. 2021 Jul 14;13(7):1362.
157. Hays LM, Black M, Prunty MP, Murthy S, van de Veerdonk FL, Annane D, Binnie A, Burrell A, Derde LP, Gordon AC, Green C. Could treatment with immunomodulatory agents

targeting IL-1, IL-6, or JAK signalling improve outcomes in patients with severe influenza pneumonia? A systematic and narrative review. HRB Open Research. 2022 Nov 28;5(77):77.
158. LI G, ZHANG L, LI Y, JIA W, ZHANG D, GAO S, ZHAO Y. Clinical and Pathological Features of Severe Influenza A H1N1 Influenza Virus Pneumonia. Tianjin Medical Journal. 2010:32-5.
159. Parikh M, Dolson G, Ramanathan V, Sangsiraprapha W. Novel H1N1-associated rhabdomyolysis leading to acute renal failure. Clinical microbiology and infection. 2010 Apr;16(4):330-2.
160. Xiang H, Song B, Zhang Y, Zhang J, Xiong J. The effectiveness of continuous renal replacement therapy in critical COVID-19 patients with cytokine release syndrome: a retrospective, multicenter, descriptive study from Wuhan, China. Aging (Albany NY). 2021 Apr 3;13(7):9243.
161. Divala TH, Fielding KL, Kandulu C, Nliwasa M, Sloan DJ, Gupta-Wright A, Corbett EL. Utility of broad-spectrum antibiotics for diagnosing pulmonary tuberculosis in adults: a systematic review and meta-analysis. The Lancet Infectious Diseases. 2020 Sep 1;20(9):1089-98.
162. Berlet T, Etter R, Fehr T, Berger D, Sendi P, Merz TM. Sonographic patterns of lung consolidation in mechanically ventilated patients with and without ventilator-associated pneumonia: a prospective cohort study. Journal of critical care. 2015 Apr 1;30(2):327-33.
163. Berlet T, Etter R, Fehr T, Berger D, Sendi P, Merz TM. Sonographic patterns of lung consolidation in mechanically ventilated patients with and without ventilator-associated pneumonia: a prospective cohort study. Journal of critical care. 2015 Apr 1;30(2):327-33.
164. Hwang S Y, Shin T G, Jo I J, et al. Neutrophil-to-lymphocyte ratio as a prognostic marker in critically-ill septic patients[J]. The American journal of emergency medicine, 2017, 35(2): 234-239.
165. Wittermans E, van de Garde E M W, Voorn G P, et al. Neutrophil count, lymphocyte count and neutrophil-to-lymphocyte ratio in relation to response to adjunctive dexamethasone treatment in community-acquired pneumonia[J]. European journal of internal medicine, 2022, 96: 102-108.
166. Shi JX, Shao XR, Wu P, Wang X, Sheng K, Chen QC, Jin F. Clinical features of influenza-associated pulmonary aspergillosis: a retrospective multicenter cohort study. Frontiers in Cellular and Infection Microbiology. 2025 Oct 15;15:1648547.
167. Shusterman E, Prozan L, Ablin JN, Weiss-Meilik A, Adler A, Choshen G, Kehat O. Neutrophil-to-lymphocyte ratio trend at admission predicts adverse outcome in hospitalized respiratory syncytial virus patients. Heliyon. 2023 Jun 1;9(6).
168. Al Mutair A, Daniyal M, Alkubati SA, Albaqawi H, Alrasheeday AM, Alshammari B, Alsaleh K, Mottershead R, Alyami H, Alharbi HF, Al-Omari A. Comparative effectiveness of anti-inflammatory and antiviral therapies on NLR and survival outcomes in severe COVID-19: multicenter retrospective study. PeerJ. 2025 Sep 4;13:e20003.
169. Yang L, Gao C, He Y, Wang X, Yang L, Guo S, Chen J, He S, Sun Y, Gao Y, Guo Q. The Neutrophil-to-Lymphocyte Ratio is Associated with the Requirement and the Duration of Invasive Mechanical Ventilation in Acute Respiratory Distress Syndrome Patients: A Retrospective Study. Canadian Respiratory Journal. 2022;2022(1):1581038.
170. Moisa E, Corneci D, Negoita S, Filimon CR, Serbu A, Negutu MI, Grintescu IM. Dynamic changes of the neutrophil-to-lymphocyte ratio, systemic inflammation index, and derived neutrophil-to-lymphocyte ratio independently predict invasive mechanical ventilation need and death in critically ill COVID-19 patients. Biomedicines. 2021 Nov 10;9(11):1656.
171. Burlacu A, Namolovan CA, Brinza C, Covic A, Floria M, Voroneanu L, Covic A. Neutrophil-to-lymphocyte ratio (NLR)—Independent prognostic marker of renal function decline in chronic kidney disease: A systematic review and meta-analysis. Journal of Clinical Medicine. 2025 Sep 26;14(19):6822.
172. Lee J, Song J, Kim SG, Yun D, Kang MW, Kim DK, Oh KH, Joo KW, Kim YS, Han SS, Kim YC. Mortality associated with the neutrophil-lymphocyte ratio in septic acute kidney injury requiring continuous renal replacement therapy. Kidney Research and Clinical Practice. 2024 Feb 8;43(3):337.
173. Nunez JI, Uehara M, Patel SR, Forest SJ, Rochlani Y, Madan S, Sims DB, Mellas N, Ashley JE, Rahmanian M, Carlese A. Changes in neutrophil-to-lymphocyte ratio during venoarterial

extracorporeal membrane oxygenation. ASAIO Journal. 2025 Jul 1;71(7):528-35.
174. Hedberg P, Johansson N, Ternhag A, Abdel-Halim L, Hedlund J, Nauclér P. Bacterial co-infections in community-acquired pneumonia caused by SARS-CoV-2, influenza virus and respiratory syncytial virus. BMC infectious diseases. 2022 Jan 31;22(1):108.
175. Wang Y, Cui X, Tian R, Wang P. The epidemiological characteristics of invasive pulmonary aspergillosis and risk factors for treatment failure: a retrospective study. BMC Pulmonary Medicine. 2024 Nov 8;24(1):559.
176. Torres A, Sibila O, Ferrer M, et al. Effect of corticosteroids on treatment failure among hospitalized patients with severe community-acquired pneumonia and high inflammatory response: a randomized clinical trial[J]. Jama, 2015, 313(7): 677-686.
177. Soriano Puig A, Monforte V, Camprubí-Rimblas M, et al. Biomarker-guided use of corticosteroids in pneumonia[J]. Pneumonia, 2026, 18(1): 4.
178. Braig D, Nero TL, Koch HG, Kaiser B, Wang X, Thiele JR, Morton CJ, Zeller J, Kiefer J, Potempa LA, Mellett NA. Transitional changes in the CRP structure lead to the exposure of proinflammatory binding sites. Nature communications. 2017 Jan 23;8(1):14188.
179. O'Driscoll LS, Martin-Loeches I. Management of severe influenza. Seminars in Respiratory and Critical Care Medicine. 2021 Dec;42(6):771-787.
180. Ngwa DN, Pathak A, Agrawal A. IL-6 regulates induction of C-reactive protein gene expression by activating STAT3 isoforms. Molecular immunology. 2022 Jun 1;146:50-6.
181. Ridker PM, Rane M. Interleukin-6 signaling and anti-interleukin-6 therapeutics in cardiovascular disease. Circulation research. 2021 May 28;128(11):1728-46.
182. Levinson T, Wasserman A, Shenhar-Tsarfaty S, Halutz O, Shapira I, Zeltser D, Rogowski O, Berliner S, Ziv-Baran T. Comparative analysis of CRP as a biomarker of the inflammatory response intensity among common viral infections affecting the lungs: COVID-19 versus influenza A, influenza B and respiratory syncytial virus. Clinical and Experimental Medicine. 2023 Dec;23(8):5307-13.
183. Sun W, Luo Z, Jin J, Cao Z, Ma Y. The neutrophil/lymphocyte ratio could predict noninvasive mechanical ventilation failure in patients with acute exacerbation of chronic obstructive pulmonary disease: a retrospective observational study. International Journal of Chronic Obstructive Pulmonary Disease. 2021 Aug 5:2267-77.
184. Ishibuchi K, Fukasawa H, Kaneko M, Yasuda H, Furuya R. Elevation of creatine kinase is associated with acute kidney injury in hospitalized patients infected with seasonal influenza virus. Clinical and Experimental Nephrology. 2021 Apr;25(4):394-400.
185. Grasso S, Terragni P, Birocco A, Urbino R, Del Sorbo L, Filippini C, Mascia L, Pesenti A, Zangrillo A, Gattinoni L, Ranieri VM. ECMO criteria for influenza A (H1N1)-associated ARDS: role of transpulmonary pressure. Intensive care medicine. 2012 Mar;38(3):395-403.
186. Zangrillo A, Biondi-Zoccai G, Landoni G, Frati G, Patroniti N, Pesenti A, Pappalardo F. Extracorporeal membrane oxygenation (ECMO) in patients with H1N1 influenza infection: a systematic review and meta-analysis including 8 studies and 266 patients receiving ECMO. critical care. 2013 Feb 13;17(1):R30.
187. Simon L, Gauvin F, Amre DK, Saint-Louis P, Lacroix J. Serum procalcitonin and C-reactive protein levels as markers of bacterial infection: a systematic review and meta-analysis. Clinical infectious diseases. 2004 Jul 15;39(2):206-17.
188. Gu Q, Yuan K, Wei J, Yoon CH, Danielsen AS, Luk A, Eyre DW, Walker AS. Interplay between C-reactive protein responses and antibiotic prescribing in people with suspected infection. BMC Infectious Diseases. 2025 Aug 5;25(1):987.
189. Garnacho-Montero J, León-Moya C, Gutiérrez-Pizarraya A, et al. Clinical characteristics, evolution, and treatment-related risk factors for mortality among immunosuppressed patients with influenza A (H1N1) virus admitted to the intensive care unit[J]. Journal of critical care, 2018, 48: 172-177.
190. Li W, Chen G, Lin F, Yang H, Cui Y, Lu R, Song C, Li H, Li Y, Pan P. A score for predicting invasive pulmonary aspergillosis in immunocompetent critically ill patients. European Journal of Clinical Investigation. 2023 Jul;53(7):e13985.

191. Chong WH, Saha BK, Neu KP. Comparing the clinical characteristics and outcomes of COVID-19-associate pulmonary aspergillosis (CAPA): a systematic review and meta-analysis. Infection. 2022 Feb;50(1):43-56.
192. Wu J, Shen J, Han Y, Qiao Q, Dai W, He B, Pang R, Zhao J, Luo T, Guo Y, Yang Y. Upregulated IL-6 indicates a poor COVID-19 prognosis: a call for tocilizumab and convalescent plasma treatment. Frontiers in immunology. 2021 Mar 4;12:598799.
193. Lavillegrand JR, Garnier M, Spaeth A, Mario N, Hariri G, Pilon A, Berti E, Fieux F, Thietart S, Urbina T, Turpin M. Elevated plasma IL-6 and CRP levels are associated with adverse clinical outcomes and death in critically ill SARS-CoV-2 patients: inflammatory response of SARS-CoV-2 patients. Annals of Intensive Care. 2021 Jan 13;11(1):9.
194. Windisch W, Walterspacher S, Siemon K, Geiseler J, Sitter H. Guidelines for non-invasive and invasive mechanical ventilation for treatment of chronic respiratory failure. Pneumologie. 2010 Oct;64(10):640-52.
195. Awadallah MF, Taha AT, Sarhan TS. Cardiorespiratory changes and outcome during noninvasive and invasive mechanical ventilation in ARDS: a comparative study. Research and Opinion in Anesthesia & Intensive Care. 2021 Jan 1;8(1):6-12.
196. Sitbon A, Darmon M, Geri G, Jaubert P, Lamouche-Wilquin P, Monet C, Le Fèvre L, Baron M, Harlay ML, Bureau C, Joannes-Boyau O. Accuracy of clinicians' ability to predict the need for renal replacement therapy: a prospective multicenter study. Annals of Intensive Care. 2022 Oct 15;12(1):95.
197. Rozencwajg S, Fraser J, Montero S, Combes A, Schmidt M. To be or not to be on ECMO: can survival prediction models solve the question?. Critical Care and Resuscitation. 2017 Oct 1;19:21-8.
198. Giordano L, Francavilla A, Bottio T, Dell'Amore A, Gregori D, Navalesi P, Lorenzoni G, Baldi I. Predictive models in extracorporeal membrane oxygenation (ECMO): a systematic review. Systematic Reviews. 2023 Mar 15;12(1):44.
199. Vaquero-Roncero LM, Sánchez-Barrado E, Escobar-Macias D, Arribas-Pérez P, Gonzalez-Porras JR, Bermejo-Martín JF, Doncel C, Bastida JM, Hernández-Valero A, Montoto CJ, Sánchez-Crespo J. C-Reactive protein and SOFA score as early predictors of critical care requirement in patients with COVID-19 pneumonia in Spain. medRxiv. 2020 May 24:2020-05.
200. Wallis R S, O'Garra A, Sher A, et al. Host-directed immunotherapy of viral and bacterial infections: past, present and future[J]. Nature Reviews Immunology, 2023, 23(2): 121-133.
201. Torres A, Sibila O, Ferrer M, et al. Effect of corticosteroids on treatment failure among hospitalized patients with severe community-acquired pneumonia and high inflammatory response: a randomized clinical trial[J]. Jama, 2015, 313(7): 677-686.
202. Feys S, Cardinali-Benigni M, Lauwers H M, et al. Profiling bacteria in the lungs of patients with severe influenza versus COVID-19 with or without aspergillosis[J]. American Journal of Respiratory and Critical Care Medicine, 2024, 210(10): 1230-1242.
203. Patton M J, Orihuela C J, Harrod K S, et al. COVID-19 bacteremic co-infection is a major risk factor for mortality, ICU admission, and mechanical ventilation[J]. Critical Care, 2023, 27(1): 34.
204. Rouze A, Povoa P, Martin-Loeches I, et al. Early bacterial co-infections and ventilator-associated lower respiratory tract infections among intubated patients during the first and second COVID-19 waves: a European comparative cohort study[J]. Respiratory Research, 2025, 26(1): 83.
205. Winner K M, Chanderraj R, Nuppnau M, et al. Antianaerobic Antibiotics, Gut Microbiota, and Sepsis-associated Acute Kidney Injury[J]. American journal of respiratory and critical care medicine, 2026, 212(2): 314-326.
206. Mariano F, Depetris N, Malvasio V, et al. Coupled-plasma filtration and adsorption for severe burn patients with septic shock and acute kidney injury treated with renal replacement therapy[J]. Burns, 2020, 46(1): 190-198.
207. Kubler A, Maguire F, Sidebotham D. Venovenous extracorporeal membrane oxygenation for treating very severe pneumonia in aotearoa New Zealand: a 16-year experience[J]. The New

Zealand Medical Journal (Online), 2021, 134(1542): 56-66.

208. Rouze A, Povoa P, Martin-Loeches I, et al. Early bacterial co-infections and ventilator-associated lower respiratory tract infections among intubated patients during the first and second COVID-19 waves: a European comparative cohort study[J]. Respiratory Research, 2025, 26(1): 83.

209. Meng M, Chu Y, Zhang S, et al. Corticosteroid treatment in severe patients with SARS-CoV-2 and chronic HBV co-infection: a retrospective multicenter study[J]. BMC Infectious Diseases, 2022, 22(1): 891.

210. Ahmadikia K, Hashemi S J, Khodavaisy S, et al. The double-edged sword of systemic corticosteroid therapy in viral pneumonia: A case report and comparative review of influenza-associated mucormycosis versus COVID-19 associated mucormycosis[J]. Mycoses, 2021, 64(8): 798-808.

211. Pervaiz A, Soubani A O. Virus-associated pulmonary aspergillosis: A rising challenge in respiratory infections[J]. The American Journal of the Medical Sciences, 2026,371(4):313-319.

212. Sarikas C, Yanik T, Yesiler F I, et al. P. 055: Cytomegalovirus, aspergillus, influenza and XDR bacterial pneumoniae coinfection in kidney transplant patients: A case report[J]. Transplantation, 2024, 108(9S).

213. Nejabat M, Motamedifar M, Fard S A, et al. Immune Dysregulation in HIV and COVID-19 Co-infection: Therapeutic Implications[J]. Immunity, Inflammation and Disease, 2025, 13(3): e70164.

214. Minkove S J, Geiger G, Llibre J M, et al. Clinical outcomes after IL-6 blockade in patients with COVID-19 and HIV: a case series[J]. AIDS research and therapy, 2022, 19(1): 6.

215. Do Rego H, Dessajan J, Le Hingrat Q, et al. Impact of respiratory viruses detection on outcomes in ventilated nosocomial pneumonia: an exposed/unexposed study[J]. Annals of Intensive Care, 2025, 15(1): 172.

216. Yuan Y, Guan W D, Jiang H M, et al. Veno-venous extracorporeal membrane oxygenation for patient with a history of open cholecystectomy and acute respiratory distress syndrome caused by coinfection of avian influenza A (H7N9) and Epstein-Barr virus[J]. Chinese Medical Journal, 2019, 132(11): 1363-1364

217. Ray P E, Moudgil A, Sinha A. Viral infections and the kidney[M]//Pediatric Nephrology. Cham: Springer International Publishing, 2022: 707-733.

218. Alosaimi B, Naeem A, Hamed M E, et al. Influenza co-infection associated with severity and mortality in COVID-19 patients[J]. Virology journal, 2021, 18(1): 127.

219. Yuan Y, Guan W D, Jiang H M, et al. Veno-venous extracorporeal membrane oxygenation for patient with a history of open cholecystectomy and acute respiratory distress syndrome caused by coinfection of avian influenza A (H7N9) and Epstein-Barr virus[J]. Chinese Medical Journal, 2019, 132(11): 1363-1364.

220. Pervaiz A, Soubani A O. Virus-associated pulmonary aspergillosis: A rising challenge in respiratory infections[J]. The American Journal of the Medical Sciences, 2025.

221. Vanderbeke L, Janssen N A F, Bergmans D C J J, et al. Posaconazole for prevention of invasive pulmonary aspergillosis in critically ill influenza patients (POSA-FLU): a randomised, open-label, proof-of-concept trial[J]. Intensive care medicine, 2021, 47(6): 674-686.

222. Rose-John S, Winthrop K, Calabrese L. The role of IL-6 in host defence against infections: immunobiology and clinical implications. Nature Reviews Rheumatology. 2017 Jul;13(7):399-409.

223. Pantaleón García J, Wurster S, Albert ND, Bharadwaj U, Bhoda K, Kulkarni VK, Ntita M, Rodríguez Carstens P, Burch-Eapen M, Covarrubias López D, Foncerrada Lizaola J. Immunotherapy with nebulized pattern recognition receptor agonists restores severe immune paralysis and improves outcomes in mice with influenza-associated pulmonary aspergillosis. Mbio. 2025 May 14;16(5):e04061-24.

224. Li X, Lau SK, Woo PC. Fungal infection risks associated with the use of cytokine antagonists and immune checkpoint inhibitors. Experimental Biology and Medicine. 2020 Jul;245(13):1104-14.

225. Razazi K, Arrestier R, Haudebourg AF, Benelli B, Carteaux G, Decousser JW, Fourati S, Woerther PL, Schlemmer F, Charles-Nelson A, Botterel F. Risks of ventilator-associated

pneumonia and invasive pulmonary aspergillosis in patients with viral acute respiratory distress syndrome related or not to Coronavirus 19 disease. Critical Care. 2020 Dec 18;24(1):699.
226. Chen F, Qasir D, Morris AC. Invasive pulmonary aspergillosis in hospital and ventilator-associated pneumonias. InSeminars in respiratory and critical care medicine 2022 Apr (Vol. 43, No. 02, pp. 234-242). Thieme Medical Publishers, Inc..
227. Torres A, Martin-Loeches I. Invasive pulmonary aspergillosis in ventilator-associated pneumonia: the hidden enemy?. American Journal of Respiratory and Critical Care Medicine. 2020 Oct 15;202(8):1071-3.
228. Ledoux MP, Guffroy B, Nivoix Y, Simand C, Herbrecht R. Invasive pulmonary aspergillosis. InSeminars in respiratory and critical care medicine 2020 Feb (Vol. 41, No. 01, pp. 080-098). Thieme Medical Publishers.
229. Gandhi BV, Bahadur MM, Dodeja H, Aggrwal V, Thamba A, Mali M. Systemic fungal infections in renal diseases. Journal of postgraduate medicine. 2005 Oct 1;51(Suppl 1):S30-6.
230. Lahmer T, Peçanha-Pietrobom PM, Schmid RM, Colombo AL. Invasive fungal infections in acute and chronic liver impairment: a systematic review. Mycoses. 2022 Feb;65(2):140-51."
231. Chong WH, Saha BK, Tan CK. Clinical characteristics and outcomes of influenza-associated pulmonary aspergillosis among critically ill patients: a systematic review and meta-analysis. Journal of Hospital Infection. 2022 Feb 1;120:98-109.
232. Thomas-Rüddel DO, Schlattmann P, Pletz M, Kurzai O, Bloos F. Risk factors for invasive Candida infection in critically ill patients: a systematic review and meta-analysis[J]. Chest. 2022,161(2):345-55.
233. Drummond RA, Desai JV, Ricotta EE, Swamydas M, Deming C, Conlan S, Quinones M, Matei-Rascu V, Sheriff L, Lecky D, Lee CC. Long-term antibiotic exposure promotes mortality after systemic fungal infection by driving lymphocyte dysfunction and systemic escape of commensal bacteria[J]. Cell host & microbe. 2022,30(7):1020-33.
234. Garcia-Olivé I, Fiz J A, Sanz-Santos J, et al. Association of influenza-like illness and climatic parameters with hemoptysis[J]. Respiratory Care, 2014, 59(11): 1726-1730.
235. Chu S E, Seak C J, Su T H, et al. Prognostic accuracy of SIRS criteria and qSOFA score for in-hospital mortality among influenza patients in the emergency department[J]. BMC Infectious Diseases, 2020, 20(1): 385.
236. Fullana Barceló M I, Artigues Serra F, Millan Pons A R, et al. Analysis of viral pneumonia and risk factors associated with severity of influenza virus infection in hospitalized patients from 2012 to 2016[J]. BMC Infectious Diseases, 2024, 24(1): 302.
237. Soultan K M, Kewalramani A, Bradbury A, et al. Clinical impact of influenza a coinfection on staphylococcus aureus pneumonia during the 2024-2025 influenza season in New York City[J]. International Journal of Infectious Diseases, 2025: 108072.
238. Al-Dorzi H M, Alsafwani Z A, Alsalahi E, et al. Patients with influenza admitted to a tertiary-care hospital in Riyadh between 2018 and 2022: characteristics, outcomes and factors associated with ICU admission and mortality[J]. BMC Pulmonary Medicine, 2024, 24(1): 464.
239. Moriyama M, Hugentobler W J, Iwasaki A. Seasonality of respiratory viral infections[J]. Annual review of virology, 2020, 7: 83-101.
240. Tanner H, Boxall E, Osman H. Respiratory viral infections during the 2009–2010 winter season in Central England, UK: incidence and patterns of multiple virus co-infections[J]. European journal of clinical microbiology & infectious diseases, 2012, 31(11): 3001-3006.
241. Burtseva E I, Panova A D, Kolobukhina L V, et al. Epidemic season 2021–2022: Frequency of co-infection by respiratory viral pathogens[J]. Epidemiology and Infectious Diseases, 2023, 28(2): 67-77.
242. Lu LY, Lee HM, Burke A, et al. Prevalence, risk factors, clinical features, and outcome of influenza-associated pulmonary aspergillosis in critically ill patients: a systematic review and meta-analysis. Chest. 2024, 165(3): 540-558.
243. Shi C, Shan Q, Xia J, et al. Incidence, risk factors and mortality of invasive pulmonary aspergillosis in patients with influenza: a systematic review and meta-analysis. Mycoses. 2022,

65(2):152-63.
244. Chong WH, Saha BK, Tan CK. Clinical characteristics and outcomes of influenza-associated pulmonary aspergillosis among critically ill patients: a systematic review and meta-analysis. Journal of Hospital Infection. 2022, 120: 98-109.
245. Peral J, Estella Á, Nuvials X, et al. Managing the next wave of influenza and/or SARS-CoV-2 in the ICU—practical recommendations from an expert group for CAPA/IAPA patients[J]. Journal of Fungi, 2023, 9(3): 312.
246. Martin-Loeches I, Torres A. Corticosteroids for CAP, influenza and COVID-19: when, how and benefits or harm?. European Respiratory Review, 2021, 30(159):200346.
247. Louis G, de Prost N. Corticosteroids for severe influenza pneumonia: a persistent evidence gap. Intensive Care Medicine. 2026: 1-2.
248. Lee JE, Kang DH, Ju H, et al. Epidemiology and risk factors of fungal pathogens in sepsis: a prospective nationwide multicenter cohort study. BMC Infectious Diseases. 2025, 25:331.
249. Lu LY, Lee HM, Burke A, et al. Prevalence, risk factors, clinical features, and outcome of influenza-associated pulmonary aspergillosis in critically ill patients: a systematic review and meta-analysis. Chest. 2024, 165(3): 540-558.
250. Shi C, Shan Q, Xia J, et al. Incidence, risk factors and mortality of invasive pulmonary aspergillosis in patients with influenza: a systematic review and meta-analysis. Mycoses. 2022, 65(2):152-63.
251. Chong WH, Saha BK, Tan CK. Clinical characteristics and outcomes of influenza-associated pulmonary aspergillosis among critically ill patients: a systematic review and meta-analysis. Journal of Hospital Infection. 2022, 120: 98-109.
252. Brun-Buisson C, Richard J C M, Mercat A, et al. Early corticosteroids in severe influenza A/H1N1 pneumonia and acute respiratory distress syndrome[J]. American journal of respiratory and critical care medicine, 2011, 183(9): 1200-1206.
253. Garcés H H, Lacalle A N, López L L, et al. Risk factors associated to noninvasive ventilation failure in primary influenza A pneumonia in the critical care setting[J]. Medicina Intensiva (English Edition), 2021, 45(6): 347-353.

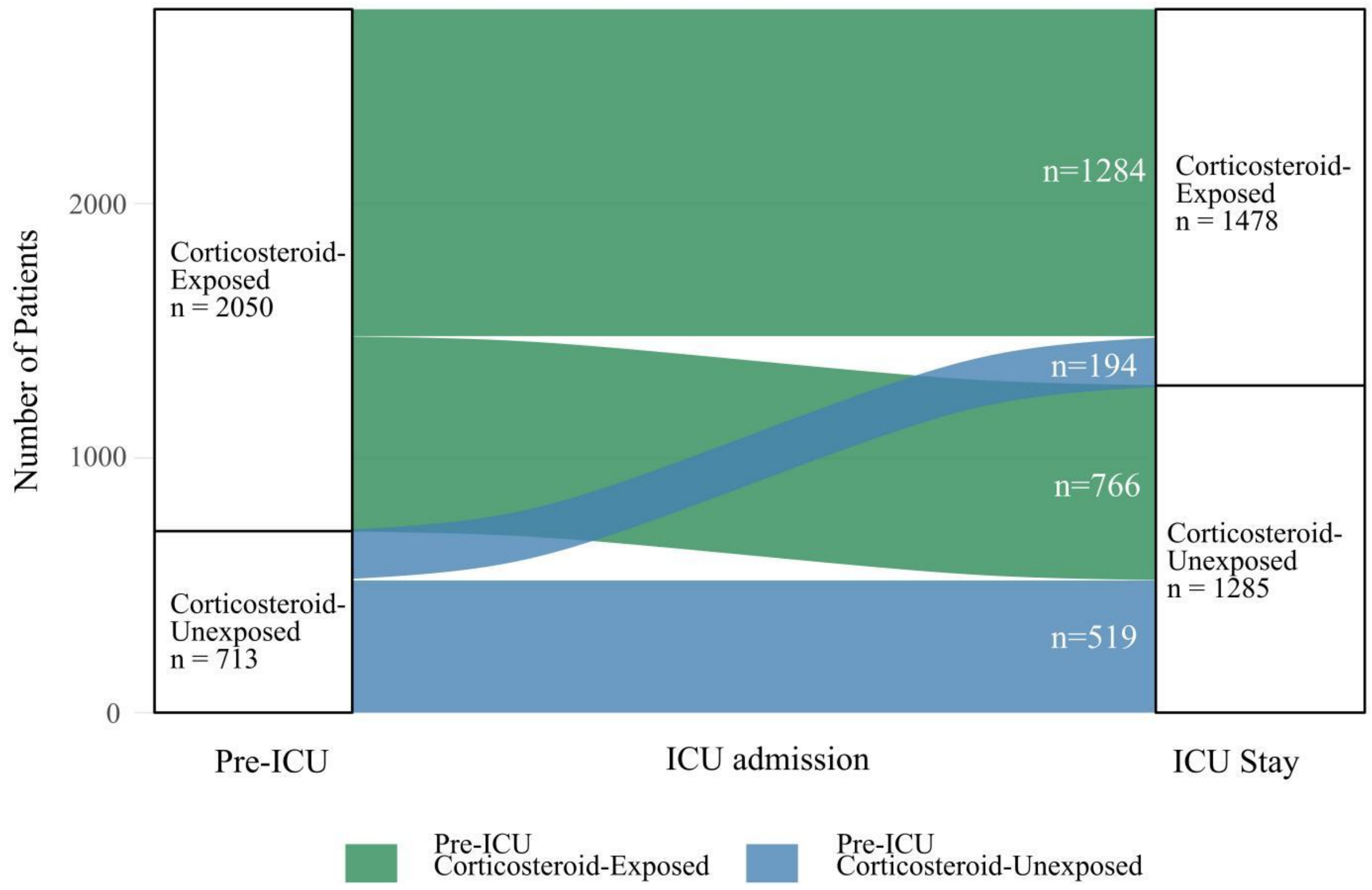

Supplementary Figure S1. Study timeline illustrating corticosteroid exposure window, ICU admission, and the intention-to-treat framework [This schematic diagram depicts the study design timeline. The 7-day pre-ICU window (left) defines corticosteroid exposure status (green) versus no exposure (blue). The moment of ICU admission is time zero, at which exposure was fixed according to the intention-to-treat principle. The ICU stay period (right) represents the follow-up duration for IAPA surveillance. The 27.2% (n=194) post-baseline crossover among initially unexposed patients is also annotated.]

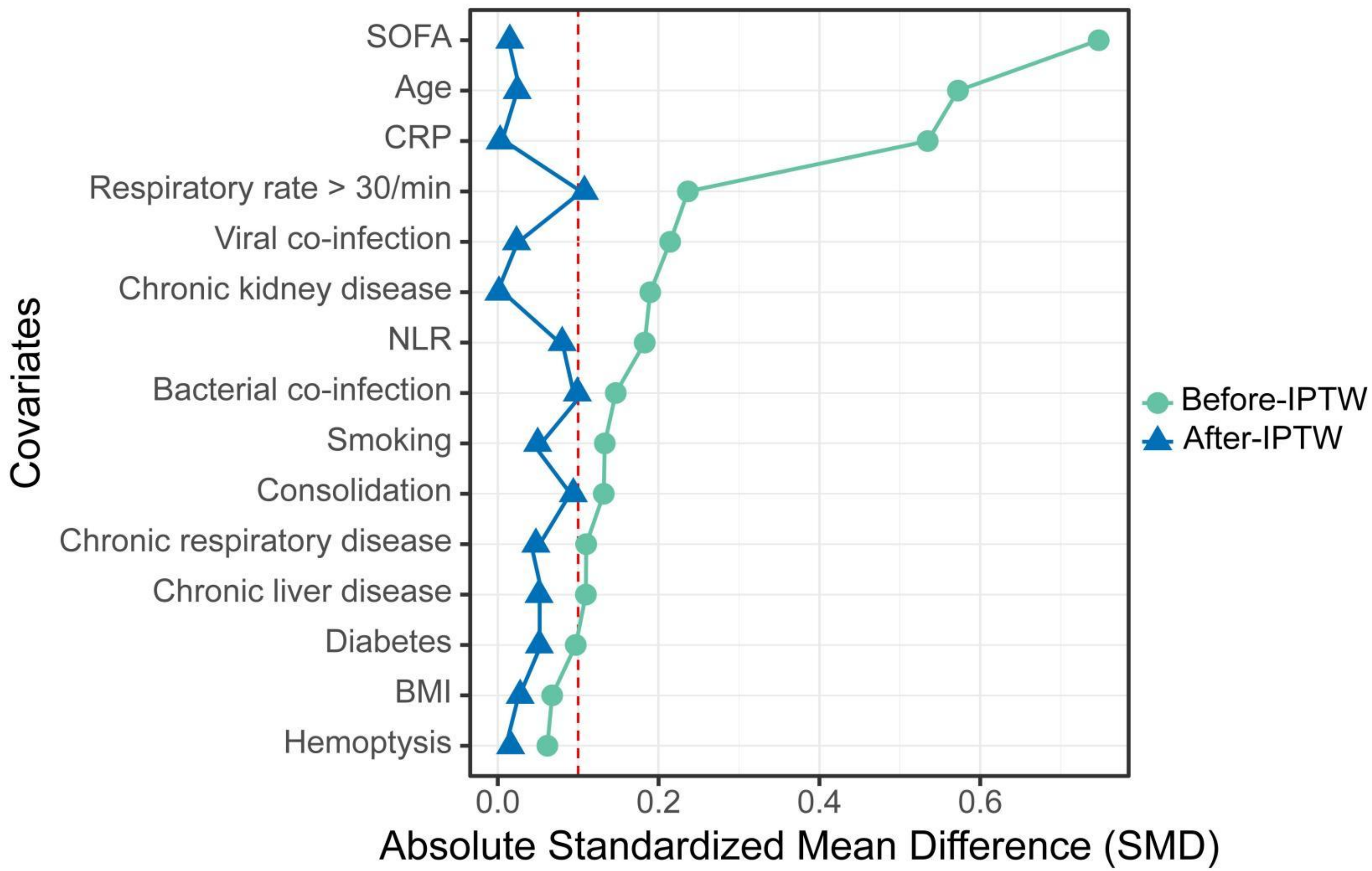

Supplementary Figure S2. Balance of DAG-derived confounders, stratified by pre-ICU corticosteroid, before and after IPTW

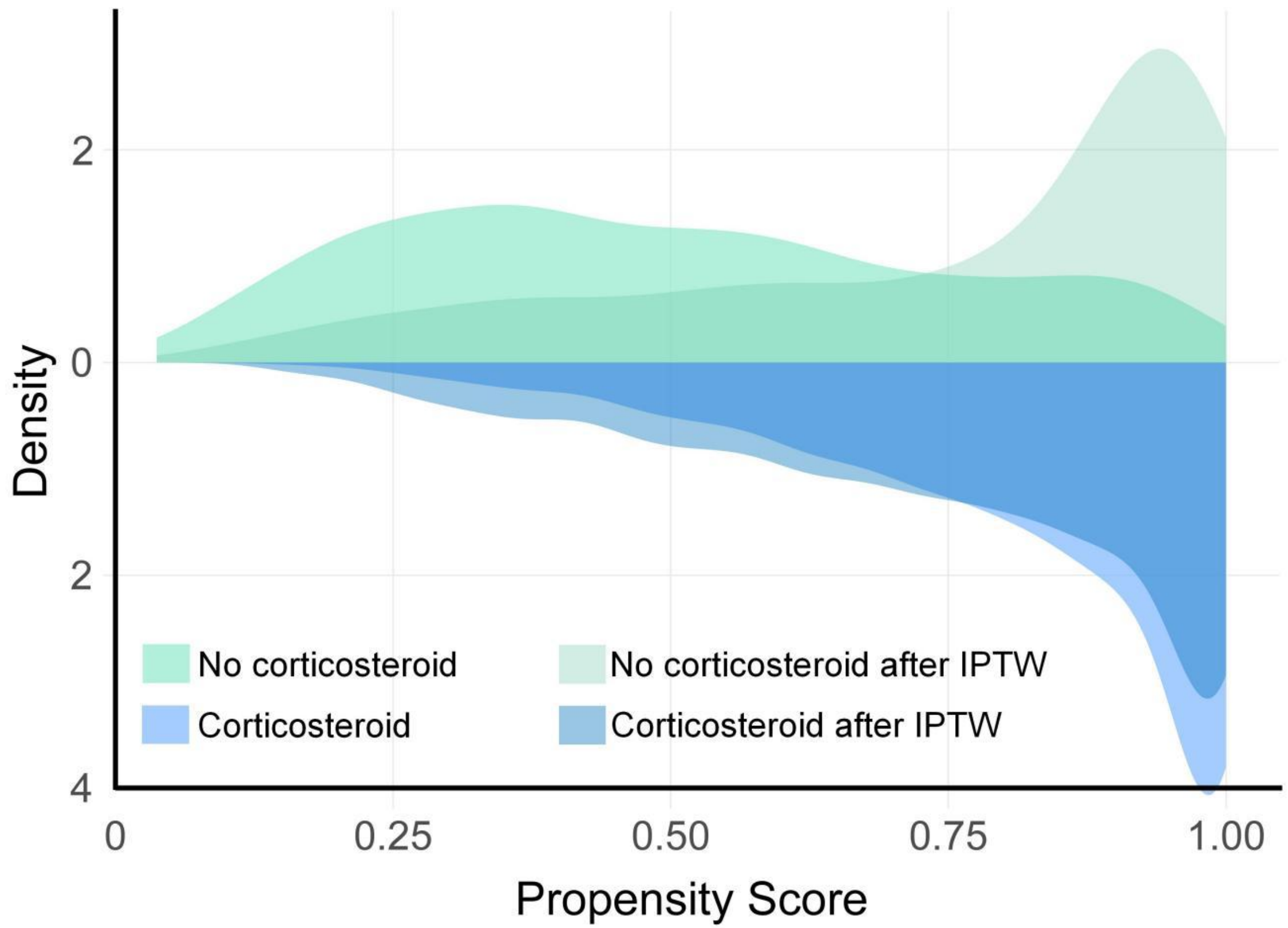

Supplementary Figure S3. The mirrored density plot of propensity scores for pre-ICU corticosteroid before and after IPTW (the area under the curve corresponds to the probability of propensity scores, estimated using kernel density smoothing method)

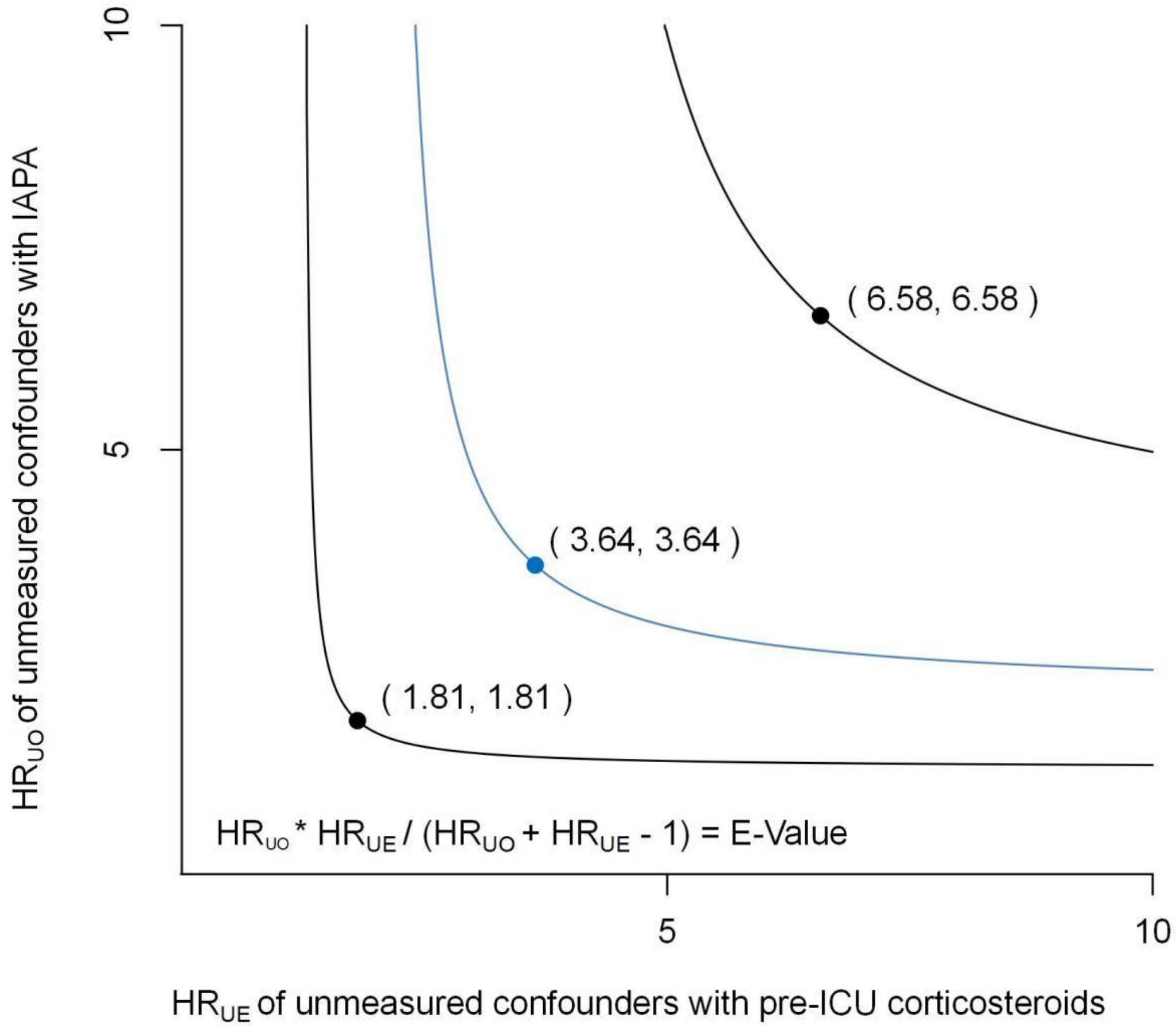

Supplementary Figure S4. E-value sensitivity analysis for unmeasured confounders (dashed lines represent upper and lower bounds)
